# Evolutionarily conserved spliceosome–exosome pathway in nuclear mRNA surveillance

**DOI:** 10.1101/gad.353594.125

**Published:** 2026-07-01

**Authors:** Daniel K. Abbas, Fabien Bonneau, Max E. Wilkinson, Steffen Schüssler, Jérôme Basquin, Elena Conti

**Affiliations:** 1 Department of Structural Cell Biology, Max Planck Institute of Biochemistry, Martinsried, Munich 82152, Germany;; 2 Structural Biology Program, Sloan Kettering Institute, Memorial Sloan Kettering Cancer Center, New York, New York 10065, USA

**Keywords:** exosome-mediated decay, mRNA export, RRP1B, quality control, TREX-2, missplicing, UAP56, cryo-EM, BioID

## Abstract

Intron-containing mRNAs are cotranscriptionally spliced and assembled into messenger ribonucleoprotein (mRNP) particles, a process monitored by surveillance pathways. Here, we combined biochemical and structural approaches to elucidate the mechanisms by which mRNPs are sorted between two opposing fates: nuclear degradation and cytoplasmic export. While the human GANP–PCID2 complex is known to connect mRNPs to nuclear export, our data indicate that the LENG8–PCID2 complex operates as an mRNP decay connector, coupling nuclear mRNPs to the RNA-degrading exosome via the PAXT adaptor complex. Both recognize the mRNP component UAP56, but LENG8–PCID2 uniquely associates with early splicing factors through a direct interaction with U1A and RRP1B. Similarly, the Thp3–Csn12 ortholog in budding yeast couples the early splicing factors Mud2–Bbp with the nuclear exosome. The spliceosome–exosome mRNP decay pathway we uncovered reveals molecular principles that remain strikingly conserved across evolution, despite the fundamental differences in splicing and decay between humans and budding yeast.

## Abstract (precis)

In this study, Abbas et al. describe the protein interactions that direct the nuclear degradation of messenger ribonucleoprotein (mRNP) particles, identifying a connector complex that couples early splicing factors with the RNA-degrading exosome. The LENG8–PCID2 complex in HEK cells and the Thp3–Csn12 complex in yeast operate as such mRNP decay connectors, presenting an evolutionarily conserved mechanism of mRNA surveillance.

In the nucleus, messenger RNAs (mRNAs) are initially transcribed by RNA polymerase II (Pol II) as immature precursor molecules that undergo extensive processing, including 5′ capping, splicing, and 3′ polyadenylation, while being cotranscriptionally packaged with mRNA-binding proteins to form messenger ribonucleoprotein (mRNP) particles. When the maturation process proceeds correctly to completion, the resulting mRNPs are exported to the cytoplasm through nuclear pore complexes (Stewart 2025). In metazoans, however, most Pol II transcription events initiated at promoters fail to produce full-length transcripts and instead terminate prematurely before reaching the poly(A) site that defines the 3′ end of the gene (Bentley 2025). Premature transcription termination can occur near the transcription start site, in the promoter-proximal pausing region, when Pol II fails to acquire the factors required to enter productive elongation (Bentley 2025). Another major class of prematurely terminated transcripts arises after the elongation checkpoint and is linked to cryptic polyadenylation sites, which are often embedded within large metazoan introns. Intronic polyadenylation and its associated cleavage event generate truncated pre-mRNAs that are retained in the nucleus and degraded (Rambout and Maquat 2024). Additionally, full-length transcripts that remain incompletely spliced can accumulate in the nucleus, where they may be stored for regulated posttranscriptional splicing or subjected to degradation (Rambout and Maquat 2024). Nuclear retention of intron-containing pre-mRNAs has also been reported in budding yeast, but the underlying mechanisms are currently thought to differ from those in metazoans (Wegener and Müller-McNicoll 2018). Whether serving to ensure quality control or to regulate transcriptional output and gene expression programs, the degradation of abortive or incompletely processed Pol II transcripts represents a major task for eukaryotic cells, a function largely carried out by the nuclear RNA exosome complexes (Rambout and Maquat 2024).

The RNA exosome is the primary 3′–5′ RNA degradation machinery in eukaryotic cells (Zinder and Lima 2017; Keidel et al. 2025). At its core, it comprises a ribonuclease complex with stably associated cofactors that transiently recruit a specific 3′–5′ nuclear helicase, which unwinds RNA and channels it into the exosome ribonuclease core for degradation (Keidel et al. 2025). In the nucleus, the RRP6, RRP47, and MPP6 cofactors recruit the MTR4 helicase (Gerlach et al. 2018; Weick et al. 2018). MTR4 in turn associates with adaptors and regulatory factors to form complexes that preferentially recognize distinct exosome substrates. In metazoans, nuclear exosome targeting (NEXT) acts on short, capped, nonpolyadenylated (pA−) RNAs (Lubas et al. 2011), whereas poly(A) tail exosome targeting (PAXT) preferentially engages longer, polyadenylated (pA+) transcripts (Meola et al. 2016). Neither NEXT nor PAXT is present in budding yeast, but PAXT is conserved in fission yeast, where its targets include meiotic and unspliced mRNAs (Lee et al. 2013; Zhou et al. 2015). In human cells, PAXT targets include intronic polyadenylated and incompletely spliced transcripts (Lee et al. 2022; Soles et al. 2025). Substrate recognition is thought to involve the PAXT scaffolding subunit ZFC3H1 and multiple features on aberrant pre-mRNPs, including U1 snRNP (an early spliceosome component that recognizes the 5′ splice site) and a downstream poly(A) site (Soles et al. 2025). The precise molecular mechanisms targeting the aberrant mRNP for degradation, however, remain unclear.

PAXT is built around the core scaffolding–helicase module, ZFC3H1–MTR4, and has been shown to associate either directly or in an RNA-dependent manner with several nuclear mRNP factors, including PABPN1 and ZC3H18 (Meola et al. 2016), PAP*γ* (Keidel et al. 2025), ZC3H3 and RBM26/27 (Keidel et al. 2025), and CBC–ARS2 (Meola et al. 2016). Mass spectrometry analysis of immunoprecipitates suggests an even broader set of associated proteins (Hein et al. 2015; Keidel et al. 2025), including the protein leukocyte receptor cluster member 8 (LENG8). The association of LENG8 with PAXT raises the hypothesis that LENG8 may function in exosome-mediated mRNA decay. LENG8 (KIAA1932) was initially identified in affinity purification experiments as part of a distinct DSS1-containing complex together with PCID2 (also known as Sac homology domain protein 1) (Baillat et al. 2005). The LENG8–PCID2–DSS1 complex shares a similar structural core with GANP–PCID2–DSS1 (hereby defined as LENG8–PCID2 and GANP–PCID2 complexes) (Clarke et al. 2025). While LENG8 is nucleoplasmic (Clarke et al. 2025), GANP is part of TREX-2, an assembly anchored at the nuclear pore complex (NPC) and involved in the export of a subset of mRNPs (Wickramasinghe et al. 2014; Stewart 2025). These observations raise the conundrum of how two structurally similar complexes may impact differently on the fate of nuclear mRNPs.

Similarly unresolved is the extent of evolutionary conservation of these pathways. In *Saccharomyces cerevisiae*, GANP and LENG8 homologs (Sac3 and Thp3, respectively) interact with distinct PCID2 paralogs to form the Sac3–Thp1–Sem1 and Thp3–Csn12–Sem1 complexes (hereafter referred to as Sac3–Thp1 and Thp3–Csn12) (Table 1). Like the human GANP–PCID2 complex, yeast Sac3–Thp1 is at the NPC and involved in mRNA export (Fischer et al. 2002; Stewart 2025). Furthermore, Sac3 was initially reported in copurifications with the conserved RNA-dependent DEAD-box ATPase Sub2. Both Sub2 and its human ortholog UAP56 are components of nuclear mRNPs (Fischer et al. 2002; Asada et al. 2023; Bonneau et al. 2023; Pacheco-Fiallos et al. 2023) and have been implicated in all aspects of mRNP biogenesis (Singh et al. 2015). These observations thus suggest evolutionary conservation of function between the GANP/Sac3 mRNA export pathways in human and budding yeast. In contrast, yeast Thp3–Csn12 has not been linked to the exosome but rather to splicing (Wilmes et al. 2008; Kuang et al. 2022). However, the association of LENG8–PCID2 with PAXT, an exosome-adaptor connected to incompletely spliced transcripts (Lee et al. 2022; Soles et al. 2025), prompted us to investigate potential functional similarities between these complexes. In this study, we elucidate the interactions and mechanisms at the biochemical and structural levels to uncover a remarkable evolutionary conservation of the molecular mechanisms that govern the fate of mRNPs in either splicing-related nuclear surveillance or export.

**Table 1.**
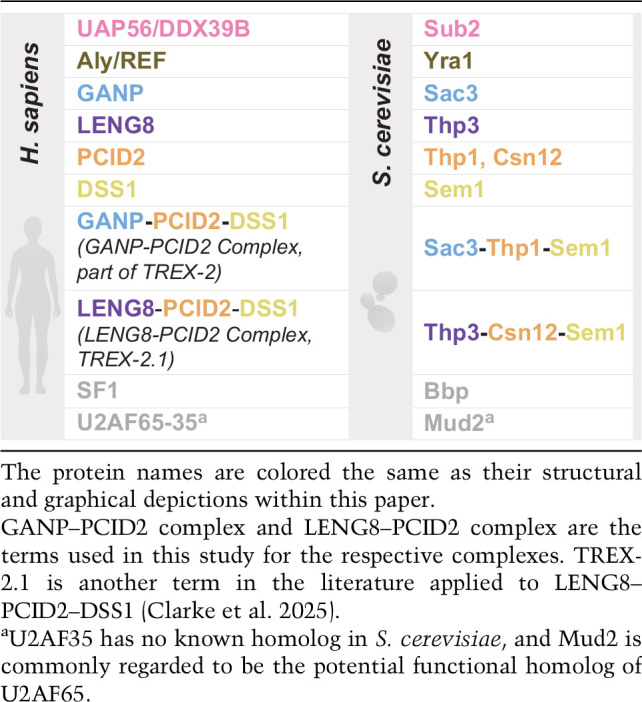
*Nomenclature of homologous* S. cerevisiae *and* Homo sapiens *proteins and complexes discussed in this study*

## Results

### Human exosome cofactor ZFC3H1 interacts directly with LENG8

**Figure 1.**
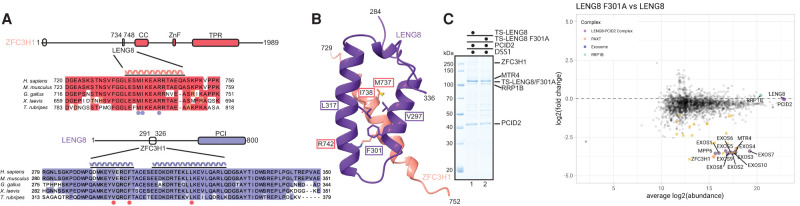
LENG8 recruits the nuclear exosome through interaction with ZFC3H1. (*A*) Domain organization and multiple sequence alignments of ZFC3H1 (salmon) and LENG8 (purple). α-Helices involved in the interaction are illustrated *above* the sequence alignments, and the specific interacting residues are indicated with colored dots. (*B*) AlphaFold-Multimer version 2.3.1 prediction depicting the LENG8 (purple) and ZFC3H1 (salmon) three helix bundle and conserved hydrophobic residues involved in the interaction. One representative prediction from 16 converging predictions is shown. (*C*) Coomassie-stained SDS gel analysis of an affinity purification performed in triplicate from HEK 293T cells (*left* panel) and plot-based enrichment analysis (*right* panel) of mass spectrometry data showing depletion of MTR4, ZFC3H1, and exosome components with the F301A point mutation in LENG8. Statistically significant fold changes at a *P*-value ≤ 0.01 are colored in gold.

To evaluate the hypothesis that LENG8 is involved in exosome-mediated mRNA decay, we first asked whether the ZFC3H1–MTR4 association with LENG8 observed in previous immunoprecipitation mass spectrometry studies (Hein et al. 2015; Keidel et al. 2025) represents a direct interaction and, if so, how it is mediated. Affinity purification from HEK 293T cells transiently coexpressing untagged MTR4 and TwinStrep (TS)–tagged ZFC3H1, followed by mass spectrometry, revealed the presence of both LENG8 and PCID2 (Supplemental Fig. S1A). DSS1 was not detectable in either the published or our mass spectrometry data, possibly due to technical limitations of the mass spectrometry protocol related to the small size and amino acid composition of the protein. AlphaFold predictions consistently converged on a putative interaction between two evolutionarily conserved motifs within the largely unstructured regions of ZFC3H1 and LENG8 (Fig. 1A,B; Supplemental Fig. S1B). In the computationally predicted model, LENG8_Z_ (ZFC3H1-binding residues 291–326) and ZFC3H1_L_ (LENG8-binding residues 734–749) interact through conserved hydrophobic residues to form a compact three-helix bundle centered at LENG8 F301 (Fig. 1A,B; Supplemental Fig. S1B).

To experimentally evaluate the computational prediction in the context of the full-length proteins, we coexpressed TS–LENG8–PCID2–DSS1, either wild type or containing a single-point mutation in LENG8 predicted to lie at the core of the three-helix bundle (F301A), and purified complexes from HEK 293T cells (Fig. 1C, left panel; Supplemental Fig. S1C,D). Assessment of the purified samples by Coomassie-stained SDS-PAGE gel indicated that MTR4 and ZFC3H1 copurified with the wild-type complex but not with the mutant (Fig. 1C, right panel,cf. lanes 1 and 2). In contrast, a prominent band that was evident in both the wild-type and LENG8 F301A mutant purifications was identified by fingerprinting mass spectrometry as ribosomal RNA processing protein 1 homolog B (RRP1B), a factor that will be discussed later. Cross-linking mass spectrometry analysis of the wild-type complex treated with bis(sulfosuccinimidyl) suberate (BS^3^) showed an interprotein cross-link between LENG8 K318 and ZFC3H1 K739, consistent with the structural prediction (Supplemental Fig. S1E–G). Finally, mass spectrometry enrichment analysis comparing the purified wild-type and mutant complexes revealed that not only ZFC3H1 and MTR4 were depleted in the LENG8 F301A mutant sample but also all the nuclear exosome core subunits (Fig. 1C, right panel; Supplemental Fig. S1D), demonstrating the crucial role of this interaction for engaging with the RNA-degradation machinery. We concluded that LENG8 recruits the nuclear exosome by interacting with the ZFC3H1 scaffolding subunit of PAXT through a small, conserved helical bundle formed within their otherwise unstructured regions.

### LENG8–PCID2 and GANP–PCID2 complexes exhibit similar binding to the DEAD-box protein UAP56

**Figure 2.**
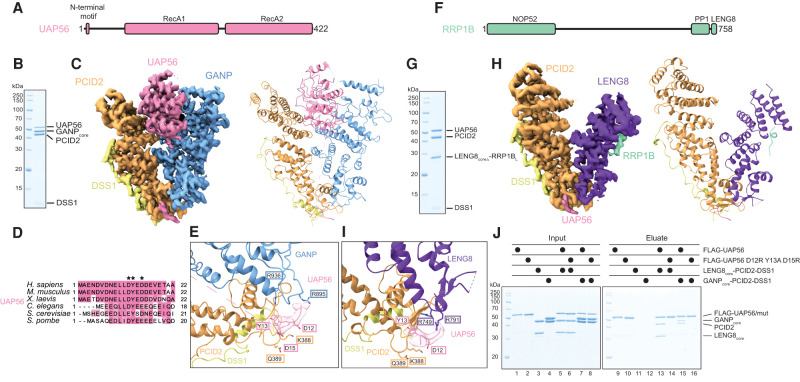
The LENG8–PCID2 and GANP–PCID2 complexes exhibit similar binding to UAP56. (*A*) Domain organization of UAP56 (pink) showing the N-terminal motif, RecA1, and RecA2. (*B*) Coomassie-stained SDS gel analysis of the reconstitution of GANP_core_–PCID2–DSS1 with UAP56 used in cryo-EM structural studies. (*C*) Single-particle cryo-EM reconstruction (*left* panel) and structural model (*right* panel) of GANP_core_–PCID2–DSS1–UAP56 at 3.0 Å. Density and model depict GANP_core_ in blue, PCID2 in orange, DSS1 in yellow, and UAP56 in pink. (*D*) Multiple sequence alignments of the UAP56 N-terminal motif involved in the interaction with the GANP–PCID2 and LENG8–PCID2 complexes. Critical interacting residues (D12, Y13, and D15) resolved structurally are marked by black stars. (*E*) Zoomed-in view of the structural model of GANP_core_–PCID2–DSS1–UAP56 focusing on the UAP56 N-terminal motif (pink) and interacting residues (UAP56 D12, Y13, and D15; PCID2 K388 and Q389; and GANP R895 and R936). Cryo-EM density for UAP56 shown in transparent pink. (*F*) Domain organization of RRP1B (green) showing the N-terminal NOP52 domain, the PP1-interacting domain (residues 682–727) (Srivastava et al. 2022), and the C-terminal LENG8 interacting domain. (*G*) Coomassie-stained SDS gel analysis of the reconstitution of LENG8_coreΔ_–RRP1B_L_–PCID2–DSS1 with UAP56 used in cryo-EM structural studies. (*H*) Single-particle cryo-EM reconstruction (*left* panel) and structural model (*right* panel) of LENG8_coreΔ_–RRP1B_L_–PCID2–DSS1–UAP56 at 2.9 Å. Density and model depict LENG8_coreΔ_ in purple, PCID2 in orange, DSS1 in yellow, UAP56 in pink, and RRP1B_L_ in green. (*I*) Zoomed-in view of the structural model of LENG8_coreΔ_–RRP1B_L_–PCID2–DSS1–UAP56 focusing on the UAP56 N-terminal motif (pink) and interacting residues (UAP56 D12, Y13, and D15; PCID2 K388 and Q389; and LENG8 R749 and R791). Cryo-EM density for UAP56 shown in transparent pink. (*J*) Coomassie-stained SDS gel analysis of FLAG-UAP56 pull-down assays. Pull-down of either the LENG8–PCID2 complex or the GANP–PCID2 complex was abolished with UAP56 containing a triple mutant of D12R Y13A D15R (cf. lanes *13*,*15* and *14*,*16*).

Having established this link to exosome-mediated decay via the PAXT complex, we sought to understand how LENG8 may engage mRNPs destined for decay. As recently reported, LENG8 and GANP assemble with PCID2–DSS1 via their core PCI domains (LENG8_core_ residues 501–800 and GANP_core_ residues 598–998), forming complexes that can bind the DEAD-box protein UAP56 in a posthydrolysis state (Clarke et al. 2025; Hohmann et al. 2026). UAP56 and its orthologs have an unstructured N-terminal region followed by the DEAD-box module with RecA1 and RecA2 domains (Fig. 2A). DEAD-box modules are well known to undergo conformational changes in response to ATP binding and hydrolysis, and these changes are in turn coupled to RNA binding and release (Ozgur et al. 2015; Hohmann et al. 2026). In contrast to previous structural studies conducted using robust chemical cross-linking (Clarke et al. 2025) or using a fusion protein of UAP56 and PCID2 (Hohmann et al. 2026), we aimed at obtaining cryo-EM reconstructions from non-cross-linked samples to gain insights into the conformation of the UAP56-bound LENG8–PCID2 and GANP–PCID2 complexes.

For the recombinant UAP56-bound GANP_core_ incubated with magnesium and ADP (Fig. 2B), we obtained a cryo-EM reconstruction at 3.0 Å resolution that displays the characteristic V-shaped architecture formed by the interaction between the helical-repeat PCI domains of GANP_core_ and PCID2, the latter of which forms a structural unit together with the small protein DSS1 (Fig. 2C; Supplemental Fig. S2A–E). A conserved motif (residues 10–15) within the N-terminal unstructured region of UAP56 binds at the convergence of the V-shaped structure, via conserved interactions primarily mediated by PCID2 (Fig. 2D,E). In the UAP56 DEAD-box module, only the RecA1 domain was resolved in the density and was positioned at the open end of the V-shaped structure, cradled between an electrostatic interface provided by GANP_core_ and a hydrophobic interface contributed by PCID2–DSS1 (Fig. 2C). Adjacent to the ADP nucleotide, we observed density for a GANP_core_ loop that has been implicated in promoting ATP turnover and facilitating RNA release (Supplemental Fig. S2F; Clarke et al. 2025; Hohmann et al. 2026).

Structural analysis of the LENG8_core_ complex required additional sample optimization, possibly reflecting greater sensitivity to air–water interface denaturation during cryo-EM grid preparation. To optimize the sample, we further trimmed LENG8 to remove a larger portion of its unstructured region (residues 550–800; LENG8_coreΔ_). We sought to enhance the stability of the complex by adding a binding partner. Useful in this context was the cross-linking mass spectrometry analysis of the TS–LENG8–PCID2–DSS1 complex purified from HEK 293T cells, as it revealed a cross-link between LENG8 and the C-terminal region of RRP1B, indicative of close spatial proximity (Supplemental Fig. S1E,H). Indeed, AlphaFold consistently predicted that LENG8 may directly interact with the RRP1B C-terminal segment (LENG8-binding residues 742–758; RRP1B_L_) (Fig. 2F; Supplemental Fig. S1I). We thus fused the LENG8_coreΔ_ with RRP1B_L_. The corresponding reconstituted LENG8_coreΔ_–RRP1B_L_–PCID2–DSS1–UAP56 complex (Fig. 2G) was amenable to cryo-EM analyses, yielding a reconstruction at ∼2.9 Å resolution (Fig. 2H; Supplemental Fig. S3). The cryo-EM structure showed that the V-shaped convergence of the LENG8_coreΔ_ complex resembles that of GANP_core_ and indeed binds the UAP56 N-terminal motif through similar interactions (Fig. 2H,I). The LENG8 PCI domain, however, is smaller as compared to that of GANP and lacks the spatial reach and the hydrophobic UAP56-binding interface characteristic of GANP (Fig. 2C,H; Supplemental Fig. S4A). Consistently, the UAP56 RecA1 domain exhibits greater flexibility in the cryo-EM density, visible only when the map was contoured at lower levels (Supplemental Fig. S4B). It thus appears that LENG8 has a weaker grip on the UAP56 DEAD-box module as compared to GANP due to structural differences in their PCI lobes (Fig. 2C,H; Supplemental Fig. S4A).

Overall, the cryo-EM reconstructions of the non-cross-linked samples revealed more flexibility in the UAP56 RecA domains as compared to the cross-linked structures (Clarke et al. 2025). The structural analyses also suggested that the UAP56 N-terminal motif, rather than the DEAD-box module, serves as the major attachment site for both LENG8–PCID2 and GANP–PCID2 complexes. In particular, a hydrophobic residue (Y13) and a cluster of acidic residues (D12, E14, and D15) at the N terminus of UAP56 nestle in a positively charged surface pocket (lined by R749 and R791 in LENG8, R895 and R936 in GANP, and K374, K388, and Q389 in PCID2) (Fig. 2E,I). Consistent with these observations, structure-based mutations in the UAP56 N-terminal motif (D12R, Y13A, and D15R) impaired the binding of full-length UAP56 to the GANP_core_ and LENG8_core_ complexes (Fig. 2J, cf. lanes 14,16, and 13,15). We concluded that the UAP56 N-terminal region is crucial for the interaction with both the LENG8–PCID2 and GANP–PCID2 complexes.

### LENG8 specifically recognizes the PP1-binding protein RRP1B

**Figure 3.**
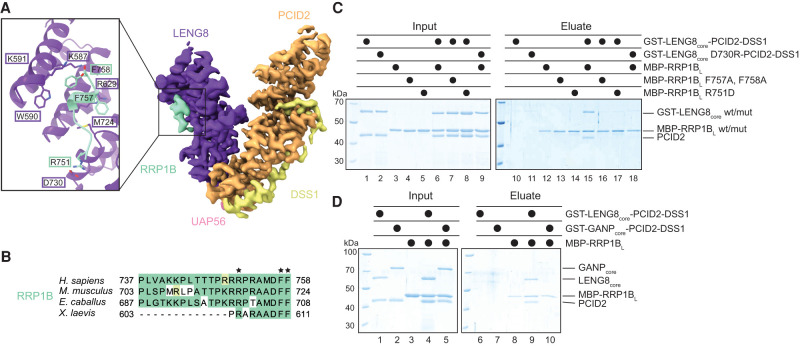
LENG8 associates with RRP1B. (*A*) Single-particle cryo-EM reconstruction (*right* panel) of LENG8_coreΔ_–RRP1B_L_–PCID2–DSS1–UAP56 and zoomed-in view of structural model (*left* panel) showing the LENG8–RRP1B interaction with key residues involved in the interaction indicated. Density and model are colored as in Figure 2H. (*B*) Multiple sequence alignments of the RRP1B C-terminal motif involved in the interaction with LENG8. Critical interacting residues (R751, F757, F758) are indicated by black stars. (*C*) Coomassie-stained SDS gel analysis of MBP-RRP1B_L_ pull-down assays. Pull-down of the LENG8–PCID2 complex was abolished with RRP1B_L_ containing either a single mutant (R751D) or a double mutant of F757A F758A, or with LENG8 containing a single mutant (D730R) (cf. lanes *15* and *16*–*18*). (*D*) Coomassie-stained SDS gel analysis of MBP-RRP1B_L_ pull-down assays. MBP-RRP1B_L_ only pulls down the LENG8–PCID2 complex but not the GANP–PCID2 complex (cf. lanes *9* and *10*).

The cryo-EM structure of the LENG8_coreΔ_–RRP1B_L_–PCID2–DSS1–UAP56 complex showed well-ordered density for RRP1B_L_, into which we could fit the AlphaFold prediction with only minor adjustments (Fig. 3A). RRP1B is a paralog of RRP1 (also known as nucleolar protein 52 kDa [NOP52]). Like RRP1, RRP1B contains a NOP52-like domain at the N terminus, shows nucleolar localization, and is involved in ribosomal RNA processing. In contrast to RRP1, however, RRP1B also has nucleoplasmic localization and has been shown to associate with chromatin (Crawford et al. 2009) and to regulate the expression of alternative mRNA isoforms by associating with splicing factors such as SRSF1 (Lee et al. 2014). A distinct feature of RRP1B is an extended C-terminal unstructured region (∼45 kDa) that is thought to confer its specific functions, including the direct interaction with protein phosphatase 1 (PP1) (in particular, isoforms *β* and *γ*) (Srivastava et al. 2022).

RRP1B_L_ encompasses the extreme C terminus of the protein, directly following the PP1-interacting segment (Srivastava et al. 2022). RRP1B_L_ docks into a prominent surface groove formed by the helical-repeat structure of LENG8 (Fig. 3A). The interaction involves conserved hydrophobic interactions (including RRP1B F757, F758 with LENG8 W590, M724) (Fig. 3A,B; Supplemental Fig. S5A). The interaction also entails electrostatic contacts (including RRP1B R751 with LENG8 D730). In addition, the carboxylate of the final C-terminal residue, F758, points to a positively charged surface patch on LENG8 (K591, K587) (Fig. 3A,B). We validated the structural analysis biochemically in pull-down assays with recombinant proteins and structure-based mutants (Fig. 3C; Supplemental Fig. S5B). While wild-type MBP-RRP1B_L_ coprecipitated wild-type LENG8_core_ complex (Fig. 3C, lane 15), substitutions of the interacting residues described above for RRP1B_L_ (Fig. 3C, lanes 16,17) and LENG8_core_ (Fig. 3C, lane 18) impaired their interaction. In line with the structural analysis, adding a C-terminal tag to an otherwise wild-type RRP1B_L_ compromised its binding to the LENG8_core_ complex (Supplemental Fig. S5C). In analogous assays, the GANP_core_ complex failed to coprecipitate with RRP1B_L_ (Fig. 3D; Supplemental Fig. S5D, cf. lanes 10 and 9). Structural superimposition of the RRP1B-bound LENG8_core_ and GANP_core_ complexes revealed that, in the latter, an extended segment folds back onto the structured lobe, occupying the corresponding surface groove formed by the helical-repeat architecture and effectively filling the surface groove intramolecularly to block RRP1B binding (Supplemental Fig. S5E). We concluded that LENG8 directly binds RRP1B at the C-terminal segment, just downstream from the PP1-binding segment (Fig. 2F).

### LENG8–RRP1B associates with mRNPs containing early splicing factors

**Figure 4.**
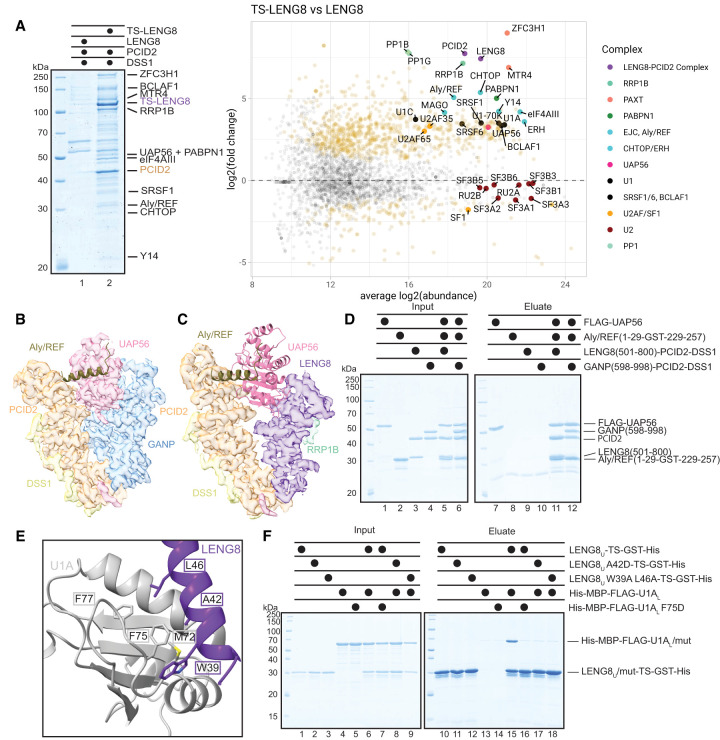
LENG8–PCID2 associates with RRP1B and mRNPs containing early splicing factors. (*A*, *left* panel) Coomassie-stained SDS gel analysis of affinity purification from HEK 293T cells overexpressing untagged LENG8, PCID2, and DSS1 or overexpressing TwinStrep (TS)-tagged LENG8, PCID2, and DSS1 with selected proteins labeled. (*Right* panel) Mass spectrometry with plot-based enrichment analysis of triplicate repeats shows high enrichment of the LENG8–PCID2 complex (purple), PAXT core complex (salmon), RRP1B (green), and PP1*β* and PP1*γ* (light blue), as well as enrichment of mRNP-associated proteins such as PABPN1 (dark green), Aly/REF, exon junction complex (EJC) proteins, CHTOP, and ERH (blue). Depicted at slightly lower levels of enrichment are UAP56 (pink) and early splicing factors (gray and yellow). Components of the U2 snRNP are shown in maroon and are not enriched. Statistically significant fold changes at a *P*-value ≤ 0.01 are colored in gold. (*B*) AlphaFold-Multimer version 2.3.1 prediction of Aly/REF–UAP56–GANP–PCID2–DSS1 (shown in cartoon representation) superimposed on the cryo-EM reconstruction of GANP_core_–PCID2–DSS1–UAP56. Only the parts of the prediction absent in the cryo-EM reconstruction are shown (Aly/REF). Interaction of the Aly/REF C-box (shown are residues 235–257) is compatible with UAP56 binding to the LENG8–PCID2 complex. Aly/REF is brown-green, UAP56 pink, LENG8 purple, PCID2 orange, and DSS1 yellow. One representative prediction from 25 converging predictions is shown. (*C*) AlphaFold-Multimer version 2.3.1 prediction of Aly/REF–UAP56–LENG8–PCID2–DSS1 (shown in cartoon representation) superimposed on the cryo-EM reconstruction of LENG8_coreΔ_–RRP1B_L_–PCID2–DSS1–UAP56. Only the parts of the prediction absent in the cryo-EM reconstruction are shown (UAP56 RecA1 and Aly/REF). Interaction of the Aly/REF C-box (shown are residues 235–257) is compatible with UAP56 binding to the LENG8–PCID2 complex. Aly/REF is brown-green, UAP56 is pink, LENG8 is purple, PCID2 is orange, DSS1 is yellow, and RRP1B is green. One representative prediction from 25 converging predictions is shown. (*D*) Coomassie-stained SDS gel analysis of FLAG-UAP56 pull-down assays of Aly/REF and the GANP–PCID2 and LENG8–PCID2 complexes. (Lanes *11*,*12*) Simultaneous pull-down of Aly/REF(1–29-GST-229–257) and the complexes was possible by FLAG-UAP56. (*E*) AlphaFold-Multimer version 2.3.1 prediction depicting the LENG8 helix (purple) and U1A RRM1 (gray) interaction and conserved hydrophobic residues (LENG8 W39, A42, and L46 and U1A M72, F75, and F77) involved in the interaction. One representative prediction from 25 converging predictions is shown. (*F*) Coomassie-stained SDS gel analysis of LENG8_U_-TS pull-down assays. Pull-down of U1A_L_ was abolished with U1A_L_ containing a single mutant (F75D) or with LENG8_U_ containing a single mutant (A42D) or a double mutant (W39A L46A) (cf. lanes *15* and *16*–*18*).

Having established that LENG8 directly binds both UAP56 and RRP1B, we next sought to identify the full complement of proteins that may directly or indirectly associate with this complex. Isolation of full-length TS–LENG8–PCID2–DSS1 expressed in HEK 293T cells resulted in a defined set of bands on a Coomassie-stained gel that, upon mass spectrometry analysis, corresponded to known mRNP components and early splicing factors (Fig. 4A, left panel). To better characterize the composition and specificity, we performed mass spectrometry–based enrichment analyses on triplicate experiments comparing TS–LENG8–PCID2–DSS1 purifications to control samples from cells expressing the untagged LENG8–PCID2 complex (Fig. 4A, right panel; Supplemental Fig. S5F,G). RRP1B had similar enrichment and fold change to LENG8 and PCID2. As expected, the PAXT core proteins MTR4 and ZFC3H1 were also highly enriched and abundant (Fig. 4A, right panel), thus reflecting robust and reproducible associations that align with our biochemical and structural analyses. The RRP1B interacting PP1*β* and PP1*γ* proteins were also highly enriched, and, in fact, we were able to identify cross-links with the RRP1B PP1-binding segment (Supplemental Fig. S5H). A slightly lower fold change was accompanied by a set of mRNP components, including the poly(A) binding protein PABPN1, subunits of the exon junction complex (eIF4AIII, Mago, and Y14), ERH–CHTOP, Aly/REF, and UAP56 (Fig. 4A, right panel), consistent with the composition identified in previous mRNP isolations and analyses (Singh et al. 2015; Bonneau et al. 2023; Pacheco-Fiallos et al. 2023). Based on previous studies (Bonneau et al. 2023; Pacheco-Fiallos et al. 2023), Aly/REF is expected to tether UAP56 to mRNPs. AlphaFold predictions suggested that Aly/REF binding should not interfere with the interactions of UAP56 with either the LENG8–PCID2 or the GANP–PCID2 complexes (Fig. 4B,C; Supplemental Fig. S5I). We biochemically confirmed the prediction in pull-down assays using FLAG-UAP56 as the bait. FLAG-UAP56 coprecipitated with both Aly/REF(1–29-GST-229–257) and either the LENG8–PCID2 or GANP–PCID2 complexes (Fig. 4D, lanes 11,12). Thus, nuclear mRNPs can in principle recruit either the LENG8–PCID2 or GANP–PCID2 complexes via the UAP56–Aly/REF interaction.

Slightly lower in terms of fold change was a cluster enriched in early splicing factors, including U1-70K, SRSF1, and SRSF6 (Fig. 4A, right panel). Since these proteins are core components and stabilizers of U1 snRNP at the 5′ splice site during the early stages of spliceosome assembly, we also explored other factors involved in these initial splicing events. At either a similar fold change or abundance as the factors above, we identified U1 snRNP components such as U1A and U1C, and the U2AF proteins that help to define the 3′ splice site for the next step in early spliceosome assembly (Martínez-Lumbreras et al. 2024), namely the recruitment of U2 snRNP. Interestingly, U2 snRNP proteins were not enriched in the purified sample (Fig. 4A, right panel).

The identification of early splicing factors in the purification of the LENG8–PCID2 complex from human cells, together with previous data showing that the PAXT subunit ZFC3H1 coimmunoprecipitated with U1 snRNP, suggested that LENG8–PCID2–DSS1 may serve as a bridge between the two (Soles et al. 2025). Using systematic AlphaFold predictions, we indeed identified a potential direct link between LENG8 and U1A. The predictions consistently converged on a possible interaction between a conserved short helix within the unstructured region of LENG8 and the first RNA-binding domain (RRM1) of U1A (Fig. 4E; Supplemental Fig. S5J). In the computationally predicted model, LENG8_U_ (U1A-binding residues 34–59) and U1A_L_ (LENG8-binding residues 1–99) interact through conserved hydrophobic residues (LENG8 W39, A42, L46 and U1A M72, F75, F77) (Supplemental Fig. S5K). We biochemically validated the putative interaction between LENG8 and U1A in pull-down assays with recombinant proteins and mutants. While wild-type LENG8_U_-TS-GST-His coprecipitated wild-type U1A_L_ (Fig. 4F, lane 15), substitutions of the interacting residues described above for U1A_L_ (Fig. 4F, lane 16) and LENG8_U_ (Fig. 4F, lanes 17,18) impaired their interaction in these assays. We concluded that a short helix in the unstructured region of LENG8 directly recognizes the U1 snRNP component U1A. Importantly, the LENG8–U1A interaction is compatible with the U1A–U1 snRNA interaction in the context of the U1 snRNP, as the RRM1 of U1A uses different surfaces to engage LENG8 and the U1 snRNA (Supplemental Fig. S5L).

Taken together, our data indicate that LENG8–PCID2 serves as a central interaction hub that connects mRNP components (via UAP56 and Aly/REF), early spliceosome components (via U1A), and decay components (via ZFC3H1).

### Yeast Thp3–Csn12 and Sac3–Thp1 complexes directly interact with Sub2

After establishing the direct molecular interactions of the human LENG8–PCID2 complex, we explored whether the orthologous *S. cerevisiae* Thp3–Csn12 complex is engaged in interactions with similar binding partners. As with the human counterpart, we included the yeast nuclear export Sac3–Thp1 complex for comparison. First, computational predictions using AlphaFold suggested that both Thp3–Csn12 and Sac3–Thp1 may bind the UAP56 ortholog Sub2 with similar interactions at its N-terminal motif (residues 9–14) (Supplemental Fig. S4C,D). Indeed, pull-down assays using recombinant Thp3–Csn12 and Sac3–Thp1 core complexes with tagged versions of the Sub2 N-terminal motif—either wild type or carrying substitutions equivalent to those used for the human ortholog UAP56 (Fig. 2J)—confirmed binding for the wild-type protein, whereas binding was impaired in the mutant (E11R Y12A D14R, Supplemental Fig. S4E). We concluded that the N-terminal region of Sub2 is required and sufficient for both Thp3–Csn12 and Sac3–Thp1 binding. Interestingly, previous in vivo studies had shown that deletion of the N-terminal motif or mutation of its central hydrophobic residue (Y12) resulted in nuclear accumulation of poly(A)^+^ RNA (Saguez et al. 2013), suggesting that Sub2 binding to either or both Thp3–Csn12 and Sac3–Thp1 is important for mRNA surveillance and/or export. Furthermore, mutations of the positively charged surface lining the interface of the Thp3–Csn12 structure had been shown to result in intron retention defects, a phenotype previously interpreted as a splicing defect due to impaired nucleic acid binding (Kuang et al. 2022). Our findings, however, suggested an alternative interpretation, that the intron retention defects associated with these mutations may instead stem from defective clearance of incompletely spliced mRNAs when Thp3–Csn12 fails to interact with Sub2. In this scenario, Thp3–Csn12 would be expected to connect the splicing and decay machineries; however, no interaction with either has been reported to date.

### Endogenous Thp3–Csn12 complex associates with early splicing factors Mud2-Bbp

**Figure 5.**
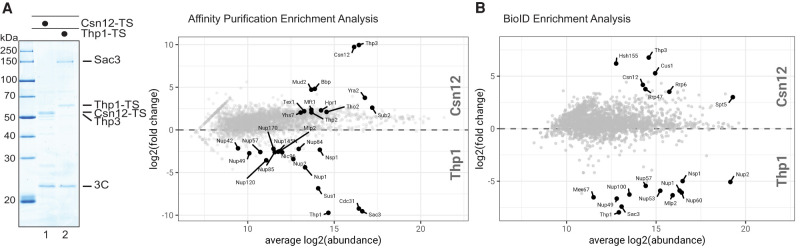
Thp3–Csn12 associates with early splicing factors and exosome subunits. (*A*, *left* panel) Coomassie-stained SDS gel analysis of affinity purification from yeast cells using ProteinA-3C-TwinStrep (TS)-tagged Csn12 or TS-tagged Thp1. (*Right* panel For Csn12, mass spectrometry plot-based enrichment analysis) of triplicate repeats shows high enrichment of Thp3, Sub2, Yra2, and the early splicing factors Bbp and Mud2. For Thp1, direct binding partners Sac3, Cdc31, and Sus1 are highly enriched, as well as nucleoporins. (*B*) Mass spectrometry plot-based BioID enrichment analysis of at least triplicate repeats comparing Csn12 and Thp1 TurboID-tagged strains. For Csn12, the direct binding partner Thp3 is enriched, along with exosome subunits Rrp6 and Rrp47. For Thp1, the direct binding partner Sac3 is enriched, along with nucleoporins.

Since Sub2 can bind to both Thp3–Csn12 and Thp1–Sac3 complexes, we endeavored to identify their additional and specific interactors that underlie their distinct functions. We first employed an affinity purification strategy using engineered yeast strains carrying endogenously integrated TS-3C-ProtA purification tags on Thp3–Csn12 and Sac3–Thp1 subunits, followed by an optimized purification procedure that we previously described for yeast nuclear mRNPs (Bonneau et al. 2023). The highest purification yields were obtained by tagging the two distinct PCID2-like subunits of these complexes, Csn12 and Thp1, resulting in visible, albeit faint, bands on Coomassie-stained gels for these rather low-abundance proteins (Fig. 5A, left panel). The respective complexes purified under native conditions were subjected to mass spectrometry proteomic characterization, with three independent replicates per sample (Supplemental Fig. S6A). The data were evaluated with a comparative enrichment analysis (Fig. 5A, right panel). Although with the limitation of not revealing shared components (e.g., Sem1), this approach is best suited to identify interactors that are uniquely associated to each complex, even in low-abundance samples.

In the case of Thp1, the enrichment analysis showed that it specifically copurified with the expected direct binding partner, Sac3, and with Cdc31 and Sus1, the two other established subunits of the TREX-2 complex (Fig. 5A, right panel; Fischer et al. 2002; Faza et al. 2009; Stewart 2025). The analysis also revealed the enrichment of nucleoporins—in line with the NPC localization of this complex (Umlauf et al. 2013). In the case of Csn12, the affinity-purified endogenous complex contained the expected direct binding partner, Thp3 (Fig. 5A, right panel). In addition, Csn12 showed a selective enrichment of mRNP proteins, in particular Sub2, Yra2 (a paralog of Yra1; the major nuclear mRNP packaging factor in yeast) (Asada et al. 2023), and THO complex proteins (Fig. 5A, right panel). We interpreted the presence of THO complex subunits as indicative of mRNPs still in the packaging stage, as THO is thought to function as a platform helping the deposition of Sub2 and Yra1 during mRNP assembly (Zenklusen et al. 2002). Notably, the Csn12 sample was enriched in Bbp (also known as Msl5) and Mud2 (Fig. 5A, right panel), two early splicing factors. Bbp and Mud2 are known to form a complex that binds the branch point sequence within an intron and cooperates with the U1 snRNP bound at the 5′ splice site to form the so-called commitment complex, the first spliceosomal assembly intermediate that sets the stage for U2 snRNP recruitment (Wang et al. 2008). Thus, the affinity purification of the Thp3–Csn12 complex revealed an association with early splicing factors involved in the initial recruitment of the spliceosome to the mRNA, reminiscent of those we had found associated with the human LENG8–PCID2 complex (Fig. 4A, right panel). The link between the yeast mRNP decay connector, Thp3–Csn12, to Mud2/Bbp is particularly compelling due to the genetic association of Mud2/Bbp with Sub2: Deletion of Mud2 or specific mutations in Bbp bypass the otherwise essential requirement for Sub2 in *S. cerevisiae* (Kistler and Guthrie 2001; Wang et al. 2008; Jacewicz et al. 2015).

### Endogenous Thp3–Csn12 complex transiently associates with nuclear exosome subunits

In contrast to the LENG8–PCID2 complex, the endogenous Thp3–Csn12 affinity purifications did not identify any RNA decay factors. Reasoning that affinity purifications primarily capture stable, long-lived interactions, while the association with the RNA-degrading machineries may be more transient in budding yeast due to the absence of a PAXT complex ortholog, we turned to proximity-dependent Biotin Identification (BioID), a method better suited for dynamic interactions. We engineered yeast strains with a C-terminal TurboID tag on either Csn12 or Thp1 (to parallel the comparison of the affinity purifications described above). We engineered an additional yeast strain with TurboID-tagged Rad52, a nuclear protein involved in a pathway that is unrelated to mRNP biogenesis (i.e., homologous recombination) (Lisby et al. 2001), to serve as a negative control in the BioID experiments. The control strain also facilitated the optimization of protocols for the efficient extraction, without degradation, of endogenously biotinylated proteins from yeast cells and for their subsequent immobilization on beads for downstream mass spectrometry analysis (detailed in the Materials and Methods; Supplemental Fig. S6B).

The results from the BioID data of the three strains (Supplemental Fig. S6C) were compared in pairwise enrichment analyses (Fig. 5B; Supplemental Fig. S6D). Comparing the Rad52 BioID data with the Csn12 BioID or Thp1 BioID data showed an enrichment of Sub2 as well as Yra1 in the latter two samples (Supplemental Fig. S6D), and in the case of Rad52, an enrichment of its known interacting factors, Rad51 and Rad59. These data, obtained in the cellular context, support the in vitro biochemical evidence for the Thp3–Csn12 and Sac3–Thp1 association with Sub2 (Supplemental Fig. S6D). We then compared the Csn12 BioID and the Thp1 BioID to better visualize their distinct interactors. In the case of Thp1, we found an enrichment of nucleoporins (including the nuclear basket component Mlp2), corroborating that the complex is embedded in the environment of the nuclear pore (Fig. 5B). In the case of Csn12, we observed an enrichment for Spt5 (a transcription elongation factor), and Cus1 and Hsh155 (factors involved in U2 snRNP recruitment [Pauling et al. 2000]), which we interpreted as the complex residing in a transcriptionally active and splicing-active microenvironment. Remarkably, the analysis showed the enrichment of Rrp6 and Rrp47 in the Csn12 BioID (Fig. 5B). The two proteins are nuclear cofactors of the RNA-degrading exosome complex, thus suggesting a missing link between the Thp3–Csn12 complex and the RNA decay machinery.

## Discussion

**Figure 6.**
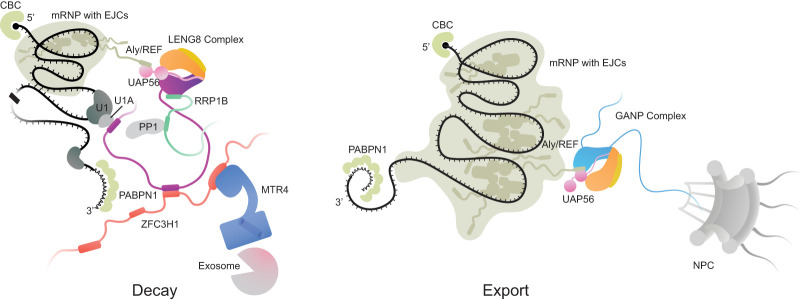
Misspliced mRNAs are targeted for degradation following recognition by the LENG8–PCID2 complex. A cartoon representation of our model shows that the LENG8–PCID2 complex (LENG8 in purple, PCID2 in orange, and DSS1 in yellow) is recruited to mRNPs (brown-green, with EJCs shown in a darker shade) via UAP56 (pink) and subsequently recognizes aberrant, misspliced mRNAs via interactions with the U1A component of the U1 snRNP (gray), resulting in exosome-mediated decay via PAXT (ZFC3H1 in salmon and MTR4 in blue). In contrast, mature, correctly processed mRNPs are associated with the nuclear pore complex-associated GANP–PCID2 complex (GANP in blue, PCID2 in orange, and DSS1 in yellow), resulting in their export from the nucleus.

In this study, we uncovered the molecular mechanisms physically linking opposite fates of nuclear mRNPs—exosome-mediated decay versus NPC-mediated export—to two structurally related complexes. Our data support a model whereby LENG8–PCID2/Thp3–Csn12 functions as a connector complex to mRNP decay, whereas GANP–PCID2/Sac3–Thp1 functions as a connector to mRNP export via the NPC (Fig. 6). In both humans and budding yeast, aberrant mRNPs containing splicing remnants are recognized by the LENG–PCID2/Thp3–Csn12 complex and are connected to the nuclear RNA-degrading exosome, while export-competent mRNPs interact with the GANP–PCID2/Sac3–Thp1 complex at the NPC as they transit to the cytoplasm. Remarkably, the core mechanisms of these two pathways have been conserved over more than a billion years of evolutionary divergence between humans and budding yeast, with adaptations reflecting species-specific differences in the features of nuclear mRNPs and their introns, as well as the compositions of exosome and spliceosome complexes.

Nuclear mRNPs are packaged cotranscriptionally by the interaction of the nascent transcript with a subset of mRNA-binding proteins. While mRNA-binding proteins associate with the 5′ cap and 3′ poly(A) tail, in budding yeast, the mRNA body is complexed with hnRNP-like proteins, with Yra1 being the predominant constituent (Asada et al. 2023). Cotranscriptional mRNP assembly also involves the THO complex and the DEAD-box ATPase Sub2 (Schuller et al. 2020). Their human counterparts are conserved, although the assembly process involves a larger set of factors. For intron-containing transcripts, the human Yra1 ortholog Aly/REF interacts directly with core components of the exon junction complex (EJC) and with additional factors such as ERH and CHTOP. Yra1 and its human ortholog Aly/REF contain long unstructured regions whose termini interact with the DEAD-box proteins Sub2/UAP56. When isolating the human LENG8–PCID2 and yeast Thp3–Csn12 complexes, we copurified these respective mRNP components (Figs. 4A, 5A). The structural and biochemical analyses of the corresponding recombinant core complexes revealed how they anchor the flexible extension of UAP56/Sub2, while the DEAD-box domains of the ATPase rest in a posthydrolysis state and in a configuration that is compatible with Yra1 and Aly/REF binding (Fig. 4B–D). These observations suggest how the mRNP decay connector complexes (LENG8–PCID2/Thp3–Csn12) can recognize the mRNPs, a mechanism that is shared by the mRNP–NPC connectors (GANP–PCID2/Sac3–Thp1) (Fig. 6). This general mRNP recognition mechanism is valid both in a model whereby UAP56/Sub2 serves as a flexible handle on the mRNP or as a clamp enclosing the mRNA within an outer shell (Clarke et al. 2025; Xie et al. 2025; Hohmann et al. 2026). Although UAP56/Sub2 participates in many direct interactions along the mRNP biogenesis pathway (Singh et al. 2015), yeast genetic data suggest that its interaction with early splicing factors (Mud2 and Bbp) constitutes its most essential function. In line with recent data that Sub2 is not required for the splicing reaction per se (Kao et al. 2025), our results point to a mechanism whereby the link between Sub2 and the mRNP decay connector functions in splicing quality control, with the removal and recycling of Bbp–Mud2 complexes that would otherwise be sequestered on stalled or aborted spliceosomes, as described below.

The mRNP decay connector complexes recognize additional features that are not present on mature ribonucleoprotein particles, namely remnants of the early splicing machinery that have failed to progress to the next step of the splicing pathway. Both the human LENG8–PCID2 and yeast Thp3–Csn12 complexes connect to the components of the first stable spliceosome assembly intermediate that identifies the 5′ splice site and branch site used for splicing (the human E complex and the yeast commitment complex), the precursors to U2 snRNP binding. Human LENG8–PCID2 directly interacts with U1A, a U1 snRNP subunit that binds at the 5′ splice-site, while yeast Thp3–Csn12 is linked with Bbp and Mud2, the subunits that bind the branch site (Figs. 4E,F, 5A). Interestingly, the mRNP decay connectors appear to have evolved to target the more robust and stable interaction in the early splicing step in each species. The branch site sequence is highly conserved in yeast and rather degenerate in humans (Taggart et al. 2017), allowing a better-defined recognition process by Bbp and its specific interactor Mud2 as compared to the human ortholog SF1 and its specific interactors U2AF65 and U2AF35. Conversely, the human U1 snRNP is about 10 times more abundant and exhibits a more stable, longer-lived interaction with the 5′ splice site compared to the yeast complex (Hansen et al. 2022). Another difference in the early splicing steps is that in humans, optimal binding of the U1 snRNP is promoted by SR proteins, such as SF2, in their phosphorylated state (Xiao and Manley 1997). Protein phosphatases have been shown to impact spliceosome assembly (Mermoud et al. 1992; Mermoud et al. 1994). The finding that the LENG8–PCID2 complex recruits a phosphatase via its interactor RRP1B raises the possibility that dephosphorylation may enable the mRNP decay connector to eventually release the U1 snRNP from the 5′ splice site to facilitate exosome-mediated decay.

Finally, the mRNP decay connector complexes are associated with the RNA-degrading exosome. In humans, LENG8–PCID2 interacts directly with the scaffolding subunit of PAXT (Fig. 1), aligning with the notion that intronic poly(A) sites are hotspots for aberrant processing in this species (Soles et al. 2025). Yeast cells lack an orthologous PAXT adaptor complex; instead, the core subunits of the nuclear exosome, Rrp6–Rrp47, are proximal interactors of Thp3–Csn12 (Fig. 5B). Based on these findings, we propose the existence of evolutionarily conserved splicing quality-control mechanisms in which the mRNP decay connector complexes LENG8–PCID2 and Thp3–Csn12, together with their interactors UAP56/Sub2 and the RNA exosome, preferentially engage the earliest stages of the splicing process. These early stages may represent a critical window in which many nascent spliceosomal assemblies are inefficient, abortive, or nonproductive and therefore require active surveillance. In this respect, exosome-mediated splicing quality control mirrors the logic of other RNA exosome-associated networks that operate at early stages of transcription, where high RNA output is coupled to rapid elimination of uncommitted or aberrant intermediates to prevent their accumulation and interference with productive gene expression. The emerging picture is that of a general nuclear surveillance strategy whereby distinct RNA exosome complexes act at early, error-prone steps across distinct mRNA biogenesis machineries to remove uncommitted intermediates.

## Materials and methods

All resources and reagents used in this study are listed in Supplemental Table S2.

### Affinity purification

For affinity purification experiments, 2 × 10^6^ HEK 293T cells were transfected with plasmids using polyethyleneimine and induced with doxycycline. Cells were transfected with untagged proteins as a negative control. Transfected cells were incubated at 37°C and harvested after 48 h. Cells were harvested by centrifuging at 800*g* for 2 min and lysed in 1 mL of lysis buffer (50 mM potassium phosphate at pH 8.0, 150 mM NaCl, 5 mM magnesium acetate, 0.1% [v/v] NP-40 supplemented with 20 µL of SUPERase RNase Inhibitor, EDTA-free cOmplete protease inhibitor cocktail) by incubating for 15 min on ice. The lysate was cleared by centrifugation and incubated with 20 µL of homemade M270-Strep-Tactin bead slurry for 1 h at 4°C. The beads were washed three times with 500 µL of lysis buffer, and bound proteins were eluted in 20 µL of SDS sample buffer or eluted with 20 mM biotin.

For cross-linking, the samples were diluted to a volume of 200 µL using lysis buffer, and 0.5 mM BS^3^ was added. After incubation for 30 min, the cross-linking reaction was quenched with 50 mM Tris (pH 7.5). The samples were then trichloroacetic acid (TCA)-precipitated.

For mass spectrometry, the samples were submitted on-bead or as TCA pellets.

### Isolation of yeast endogenous complexes

Five-hundred milliliters of yeast was grown in YPD to an absorbance of ∼1 at 600 nm, harvested by filtration, and immediately frozen in liquid nitrogen. Frozen yeast pellets were ground with a cryogenic grinder, resuspended in ice-cold purification buffer (50 mM potassium phosphate at pH 8, 0.1% NP-40), and incubated for 30 min at 4°C with Protein-G Dynabeads coated with anti-Protein A IgG. Beads were separated from the lysate with a magnet, washed in purification buffer, resuspended in purification buffer containing 3C protease, and rotated for 30 min at 4°C. 3C eluates were incubated for 30 min at 4°C with homemade epoxy-activated M270 Dynabeads conjugated to Strep-Tactin. Strep-Tactin beads were washed again in purification buffer and eluted for 30 min in purification buffer containing 10 mM biotin.

### Proximity labeling

Ten milliliters of yeast was grown in YPD to an absorbance of ∼1 at 600 nm, harvested by centrifugation, and incubated in 2 mL of 0.1 M NaOH for 5 min at room temperature to enhance extraction efficiency and reduce protein degradation (Kushnirov 2000). Total protein content was extracted by heating pelleted cells in 120 µL of SDS-PAGE loading dye for 5 min at 50°C followed by centrifugation at 18,000*g* for 5 min. Free intracellular biotin in the extract was removed using a Zeba dye and biotin removal spin column, exchanging the buffer to 50 mM Tris (pH 6.8), 2% SDS, and 500 mM NaCl. Biotin-free extract diluted twice was incubated for 24 h at 30°C with 75 µL of M270-Streptavidin Dynabeads. Beads were washed for 10 min at 40°C in 500 µL of buffer containing 50 mM Tris (pH 6.8) and 4% SDS and transferred to fresh protein LoBind tubes for elution for 10 min at 95°C with 10 µL of SDS-PAGE loading dye containing 10 mM biotin. Samples were run for 5 min at 200 V on a 4%–12% NuPAGE gel in MES buffer, stained with Protein Detective stain. Lanes were cut from the bottom of the well to just above the free Streptavidin band (13 kDa) and subjected to proteomics mass spectrometry analysis.

### Pull-downs with recombinant proteins

Pull-downs were performed by incubating bait protein with prey protein for 30 min at 4°C. Magnetic beads (anti-FLAG M2 magnetic beads for FLAG, amylose magnetic beads for MBP, and MagStrep Strep-Tactin XT beads for TwinStrep) were added, and the reaction mixture was incubated for 30 min at 4°C. The beads were washed three times, and bound proteins were eluted natively (0.4 mg/mL 3xFLAG peptide for FLAG and 25 mM maltose for MBP) or in SDS loading buffer (TwinStrep pull-downs) and visualized by SDS-PAGE.

### AlphaFold-Multimer protein structure predictions

Protein structure prediction was performed using AlphaFold version 2.3.1 (Jumper et al. 2021) with full databases. The 25 generated predicted structures were aligned using UCSF ChimeraX version 1.10 (Pettersen et al. 2021) to assess prediction convergence.

### Complex reconstitution and grid preparation for cryo-EM

Both the GANP_core_–PCID2–DSS1 complex with UAP56 and the LENG8_coreΔ_–RRP1B_L_–PCID2–DSS1 complex with UAP56 were reconstituted by mixing 60 µM complexes with 72 µM UAP56 (and 2 mM ADP in the case of GANP_core_–PCID2–DSS1) in a total volume of 25 µL in 20 mM HEPES (pH 7.5), 50 mM NaCl, 5 mM magnesium acetate, and 2 mM dithiothreitol and incubated for 30 min on ice. The samples were then loaded onto a Superdex 200 Increase 10/300 GL column and supplemented with 0.04% (v/v) n-Octyl *β*-D-glucopyranoside prior to cryo-EM grid preparation.

For cryo-EM grid preparation, 4 µL of sample was applied to R2/1 Cu200 Quantifoil grids cleaned by glow discharge with negative polarity at 30 mA for 30 sec using an EMS GloQube (Quorum). Grids were plunged into liquid ethane/propane using a Vitrobot Mark IV (Thermo Fisher Scientific) at 4°C and 95% humidity.

### Cryo-EM data acquisition and processing

High-resolution data of both the GANP_core_–PCID2–DSS1–UAP56 and the LENG8_coreΔ_–RRP1B_L_–PCID2–DSS1–UAP56 data sets were collected on an FEI Titan Krios G2 (Thermo Fisher Scientific) at 300 kV with a Gatan K3 direct electron detector in counting mode. The nominal magnification during data collection was 105,000×, corresponding to a pixel size of 0.8512 Å. Using a beam tilt-based multishot acquisition scheme in SerialEM (Schorb et al. 2019), the sample was imaged with a total exposure of 60 e^–^/Å^2^. The target defocus ranged between 0.5 and 2.0 µm. Micrographs were imported into CryoSPARC 4.7.0 (Punjani et al. 2017) for motion correction, CTF correction, particle picking, and processing in 2D and 3D (Supplemental Figs. S2, S3). For both data sets, initial particle picking was done on a subset of micrographs using the blob picker tool. The resulting 2D classes were used to train a model for the Topaz picker (Bepler et al. 2019) for particle picking on all micrographs. For both data sets, a resolution cutoff value of 0.143 gold-standard Fourier shell correlation (GSFSC) was used.

### Model building and refinement

AlphaFold predictions of GANP_core_–PCID2–DSS1–UAP56 and LENG8_coreΔ_–RRP1B_L_–PCID2–DSS1–UAP56 were fitted into their respective 3D reconstructions using UCSF ChimeraX version 1.10 (Pettersen et al. 2021). The model was manually adjusted in Coot version 0.9.8.95 (Emsley et al. 2010), and real space was refined using Phenix version 1.21.2-5419 (Afonine et al. 2018). Model quality was assessed by map to model correlation coefficients and map versus model FSCs (Supplemental Table S1).

### Data analysis and presentation

Plotting of MS enrichment data was performed with R version 4 using the Tidyverse collection of packages (Wickham et al. 2019) and the Bioconductor package proDA (Ahlmann-Eltze and Anders 2020).

### Data availability

Cryo-EM density map has been deposited in the Electron Microscopy Data Bank (EMDB) and the Protein Data Bank (PDB) under the accession numbers EMDB: 55617, PDB: 9T6L (LENG8–PCID2–DSS1 complex bound to UAP56 and RRP1B), and EMDB: 55619, PDB: 9T6N (GANP–PCID2–DSS1 complex bound to UAP56). The mass spectrometry proteomics data have been deposited to the ProteomeXchange Consortium via the PRIDE partner repository with the data set identifier PXD071678.

## Supplemental Material

Supplement 1

## References

[GAD353594ABBC2] Afonine PV, Poon BK, Read RJ, Sobolev OV, Terwilliger TC, Urzhumtsev A, Adams PD. 2018. Real-space refinement in *PHENIX* for cryo-EM and crystallography. Acta Crystallogr D Struct Biol 74: 531–544. 10.1107/S2059798318006551 29872004 PMC6096492

[GAD353594ABBC3] Ahlmann-Eltze C, Anders S. 2020. proDA: probabilistic dropout analysis for identifying differentially abundant proteins in label-free mass spectrometry. bioRxiv 10.1101/661496

[GAD353594ABBC4] Asada R, Dominguez A, Montpetit B. 2023. Single-molecule quantitation of RNA-binding protein occupancy and stoichiometry defines a role for Yra1 (Aly/REF) in nuclear mRNP organization. Cell Rep 42: 113415. 10.1016/j.celrep.2023.113415 37963019 PMC10841842

[GAD353594ABBC5] Baillat D, Hakimi MA, Näär AM, Shilatifard A, Cooch N, Shiekhattar R. 2005. Integrator, a multiprotein mediator of small nuclear RNA processing, associates with the C-terminal repeat of RNA polymerase II. Cell 123: 265–276. 10.1016/j.cell.2005.08.019 16239144

[GAD353594ABBC6] Bentley DL. 2025. Multiple forms and functions of premature termination by RNA polymerase II. J Mol Biol 437: 168743. 10.1016/j.jmb.2024.168743 39127140 PMC11649484

[GAD353594ABBC7] Bepler T, Morin A, Rapp M, Brasch J, Shapiro L, Noble AJ, Berger B. 2019. Positive-unlabeled convolutional neural networks for particle picking in cryo-electron micrographs. Nat Methods 16: 1153–1160. 10.1038/s41592-019-0575-8 31591578 PMC6858545

[GAD353594ABBC8] Bonneau F, Basquin J, Steigenberger B, Schäfer T, Schäfer IB, Conti E. 2023. Nuclear mRNPs are compact particles packaged with a network of proteins promoting RNA-RNA interactions. Genes Dev 37: 505–517. 10.1101/gad.350630.123 37399331 PMC10393194

[GAD353594ABBC9] Clarke BP, Gao S, Mei M, Xie D, Angelos AE, Vazhavilla A, Hill PS, Cagatay T, Batten K, Shay JW, 2025. Structural mechanism of DDX39B regulation by human TREX-2 and a related complex in mRNP remodeling. Nat Commun 16: 5471. 10.1038/s41467-025-60547-1 40595470 PMC12216326

[GAD353594ABBC10] Crawford NP, Yang H, Mattaini KR, Hunter KW. 2009. The metastasis efficiency modifier ribosomal RNA processing 1 homolog B (RRP1B) is a chromatin-associated factor. J Biol Chem 284: 28660–28673. 10.1074/jbc.M109.023457 19710015 PMC2781410

[GAD353594ABBC11] Emsley P, Lohkamp B, Scott WG, Cowtan K. 2010. Features and development of Coot. Acta Crystallogr D Biol Crystallogr 66: 486–501. 10.1107/S0907444910007493 20383002 PMC2852313

[GAD353594ABBC12] Faza MB, Kemmler S, Jimeno S, González-Aguilera C, Aguilera A, Hurt E, Panse VG. 2009. Sem1 is a functional component of the nuclear pore complex-associated messenger RNA export machinery. J Cell Biol 184: 833–846. 10.1083/jcb.200810059 19289793 PMC2699155

[GAD353594ABBC13] Fischer T, Strasser K, Racz A, Rodriguez-Navarro S, Oppizzi M, Ihrig P, Lechner J, Hurt E. 2002. The mRNA export machinery requires the novel Sac3p-Thp1p complex to dock at the nucleoplasmic entrance of the nuclear pores. EMBO J 21: 5843–5852. 10.1093/emboj/cdf590 12411502 PMC131087

[GAD353594ABBC14] Gerlach P, Schuller JM, Bonneau F, Basquin J, Reichelt P, Falk S, Conti E. 2018. Distinct and evolutionary conserved structural features of the human nuclear exosome complex. eLife 7: e38686. 10.7554/eLife.38686 30047866 PMC6072439

[GAD353594ABBC15] Hansen SR, White DS, Scalf M, Corrêa IR, Smith LM, Hoskins AA. 2022. Multi-step recognition of potential 5′ splice sites by the *Saccharomyces cerevisiae* U1 snRNP. eLife 11: e70534. 10.7554/eLife.70534 35959885 PMC9436412

[GAD353594ABBC16] Hein MY, Hubner NC, Poser I, Cox J, Nagaraj N, Toyoda Y, Gak IA, Weisswange I, Mansfeld J, Buchholz F, 2015. A human interactome in three quantitative dimensions organized by stoichiometries and abundances. Cell 163: 712–723. 10.1016/j.cell.2015.09.053 26496610

[GAD353594ABBC17] Hohmann U, Graf M, Tirián L, Pacheco-Fiallos B, Schellhaas U, Fin L, Handler D, Philipps AW, Riabov-Bassat D, Faraway RW, 2026. An ATP-gated molecular switch orchestrates human messenger RNA export. Nature 649: 1042–1050. 10.1038/s41586-025-09832-z 41198879 PMC12823420

[GAD353594ABBC18] Jacewicz A, Chico L, Smith P, Schwer B, Shuman S. 2015. Structural basis for recognition of intron branchpoint RNA by yeast Msl5 and selective effects of interfacial mutations on splicing of yeast pre-mRNAs. RNA 21: 401–414. 10.1261/rna.048942.114 25587180 PMC4338336

[GAD353594ABBC19] Jumper J, Evans R, Pritzel A, Green T, Figurnov M, Ronneberger O, Tunyasuvunakool K, Bates R, Žídek A, Potapenko A, 2021. Highly accurate protein structure prediction with AlphaFold. Nature 596: 583–589. 10.1038/s41586-021-03819-2 34265844 PMC8371605

[GAD353594ABBC20] Kao CY, Tsai WY, Su YL, Chung CS, Cheng SC. 2025. New mechanistic insights into prespliceosome formation-roles of DEAD-box proteins Prp5 and Sub2. RNA 31: 1901–1911. 10.1261/rna.080720.125 41027713 PMC12621592

[GAD353594ABBC21] Keidel A, Long CL, Iwasa J, Conti E. 2025. RNA-degrading exosome complexes: molecular mechanisms and structural insights. Annu Rev Cell Dev Biol 41: 505–528. 10.1146/annurev-cellbio-111822-115115 40245363

[GAD353594ABBC22] Kistler AL, Guthrie C. 2001. Deletion of MUD2, the yeast homolog of U2AF65, can bypass the requirement for sub2, an essential spliceosomal ATPase. Genes Dev 15: 42–49. 10.1101/gad.851301 11156604 PMC312603

[GAD353594ABBC23] Kuang Z, Ke J, Hong J, Zhu Z, Niu L. 2022. Structural assembly of the nucleic-acid-binding Thp3-Csn12-Sem1 complex functioning in mRNA splicing. Nucleic Acids Res 50: 8882–8897. 10.1093/nar/gkac634 35904806 PMC9410885

[GAD353594ABBC24] Kushnirov VV. 2000. Rapid and reliable protein extraction from yeast. Yeast 16: 857–860. 10.1002/1097-0061(20000630)16:9<857::AID-YEA561>3.0.CO;2-B 10861908

[GAD353594ABBC25] Lee NN, Chalamcharla VR, Reyes-Turcu F, Mehta S, Zofall M, Balachandran V, Dhakshnamoorthy J, Taneja N, Yamanaka S, Zhou M, 2013. Mtr4-like protein coordinates nuclear RNA processing for heterochromatin assembly and for telomere maintenance. Cell 155: 1061–1074. 10.1016/j.cell.2013.10.027 24210919 PMC3974623

[GAD353594ABBC26] Lee M, Dworkin AM, Gildea D, Trivedi NS, Program NCS, Moorhead GB, Crawford NP. 2014. RRP1B is a metastasis modifier that regulates the expression of alternative mRNA isoforms through interactions with SRSF1. Oncogene 33: 1818–1827. 10.1038/onc.2013.133 23604122 PMC3925194

[GAD353594ABBC27] Lee ES, Smith HW, Wolf EJ, Guvenek A, Wang YE, Emili A, Tian B, Palazzo AF. 2022. ZFC3H1 and U1-70K promote the nuclear retention of mRNAs with 5′ splice site motifs within nuclear speckles. RNA 28: 878–894. 10.1261/rna.079104.122 35351812 PMC9074902

[GAD353594ABBC28] Lisby M, Rothstein R, Mortensen UH. 2001. Rad52 forms DNA repair and recombination centers during S phase. Proc Natl Acad Sci 98: 8276–8282. 10.1073/pnas.121006298 11459964 PMC37432

[GAD353594ABBC29] Lubas M, Christensen MS, Kristiansen MS, Domanski M, Falkenby LG, Lykke-Andersen S, Andersen JS, Dziembowski A, Jensen TH. 2011. Interaction profiling identifies the human nuclear exosome targeting complex. Mol Cell 43: 624–637. 10.1016/j.molcel.2011.06.028 21855801

[GAD353594ABBC30] Martínez-Lumbreras S, Morguet C, Sattler M. 2024. Dynamic interactions drive early spliceosome assembly. Curr Opin Struct Biol 88: 102907. 10.1016/j.sbi.2024.102907 39168044

[GAD353594ABBC31] Meola N, Domanski M, Karadoulama E, Chen Y, Gentil C, Pultz D, Vitting-Seerup K, Lykke-Andersen S, Andersen JS, Sandelin A, 2016. Identification of a nuclear exosome decay pathway for processed transcripts. Mol Cell 64: 520–533. 10.1016/j.molcel.2016.09.025 27871484

[GAD353594ABBC32] Mermoud JE, Cohen P, Lamond AI. 1992. Ser/Thr-specific protein phosphatases are required for both catalytic steps of pre-mRNA splicing. Nucleic Acids Res 20: 5263–5269. 10.1093/nar/20.20.5263 1331983 PMC334330

[GAD353594ABBC33] Mermoud JE, Cohen PT, Lamond AI. 1994. Regulation of mammalian spliceosome assembly by a protein phosphorylation mechanism. EMBO J 13: 5679–5688. 10.1002/j.1460-2075.1994.tb06906.x 7988565 PMC395533

[GAD353594ABBC34] Ozgur S, Buchwald G, Falk S, Chakrabarti S, Prabu JR, Conti E. 2015. The conformational plasticity of eukaryotic RNA-dependent ATPases. FEBS J 282: 850–863. 10.1111/febs.13198 25645110

[GAD353594ABBC35] Pacheco-Fiallos B, Vorländer MK, Riabov-Bassat D, Fin L, O'Reilly FJ, Ayala FI, Schellhaas U, Rappsilber J, Plaschka C. 2023. mRNA recognition and packaging by the human transcription-export complex. Nature 616: 828–835. 10.1038/s41586-023-05904-0 37020021 PMC7614608

[GAD353594ABBC36] Pauling MH, McPheeters DS, Ares M Jr. 2000. Functional Cus1p is found with Hsh155p in a multiprotein splicing factor associated with U2 snRNA. Mol Cell Biol 20: 2176–2185. 10.1128/MCB.20.6.2176-2185.2000 10688664 PMC110834

[GAD353594ABBC37] Pettersen EF, Goddard TD, Huang CC, Meng EC, Couch GS, Croll TI, Morris JH, Ferrin TE. 2021. UCSF ChimeraX: structure visualization for researchers, educators, and developers. Protein Sci 30: 70–82. 10.1002/pro.3943 32881101 PMC7737788

[GAD353594ABBC38] Punjani A, Rubinstein JL, Fleet DJ, Brubaker MA. 2017. cryoSPARC: algorithms for rapid unsupervised cryo-EM structure determination. Nat Methods 14: 290–296. 10.1038/nmeth.4169 28165473

[GAD353594ABBC39] Rambout X, Maquat LE. 2024. Nuclear mRNA decay: regulatory networks that control gene expression. Nat Rev Genet 25: 679–697. 10.1038/s41576-024-00712-2 38637632 PMC11408106

[GAD353594ABBC40] Saguez C, Gonzales FA, Schmid M, Bøggild A, Latrick CM, Malagon F, Putnam A, Sanderson L, Jankowsky E, Brodersen DE, 2013. Mutational analysis of the yeast RNA helicase Sub2p reveals conserved domains required for growth, mRNA export, and genomic stability. RNA 19: 1363–1371. 10.1261/rna.040048.113 23962665 PMC3854527

[GAD353594ABBC41] Schorb M, Haberbosch I, Hagen WJH, Schwab Y, Mastronarde DN. 2019. Software tools for automated transmission electron microscopy. Nat Methods 16: 471–477. 10.1038/s41592-019-0396-9 31086343 PMC7000238

[GAD353594ABBC42] Schuller SK, Schuller JM, Prabu JR, Baumgärtner M, Bonneau F, Basquin J, Conti E. 2020. Structural insights into the nucleic acid remodeling mechanisms of the yeast THO-Sub2 complex. eLife 9: e61467. 10.7554/eLife.61467 33191913 PMC7744097

[GAD353594ABBC43] Singh G, Pratt G, Yeo GW, Moore MJ. 2015. The clothes make the mRNA: past and present trends in mRNP fashion. Annu Rev Biochem 84: 325–354. 10.1146/annurev-biochem-080111-092106 25784054 PMC4804868

[GAD353594ABBC44] Soles LV, Liu L, Zou X, Yoon Y, Li S, Tian L, Valdez M, Yu AM, Yin H, Li W, 2025. A nuclear RNA degradation code is recognized by PAXT for eukaryotic transcriptome surveillance. Mol Cell 85: 1575–1588.e9. 10.1016/j.molcel.2025.03.010 40187348 PMC12010247

[GAD353594ABBC45] Srivastava G, Bajaj R, Kumar GS, Gaudreau-Lapierre A, Nicolas H, Chamousset D, Kreitler D, Peti W, Trinkle-Mulcahy L, Page R. 2022. The ribosomal RNA processing 1B:protein phosphatase 1 holoenzyme reveals non-canonical PP1 interaction motifs. Cell Rep 41: 111726. 10.1016/j.celrep.2022.111726 36450254 PMC9813921

[GAD353594ABBC46] Stewart M. 2025. From transcription to export: mRNA's winding path to the cytoplasm. Trends Biochem Sci 50: 748–765. 10.1016/j.tibs.2025.06.004 40670258 PMC7619002

[GAD353594ABBC47] Taggart AJ, Lin CL, Shrestha B, Heintzelman C, Kim S, Fairbrother WG. 2017. Large-scale analysis of branchpoint usage across species and cell lines. Genome Res 27: 639–649. 10.1101/gr.202820.115 28119336 PMC5378181

[GAD353594ABBC48] Umlauf D, Bonnet J, Waharte F, Fournier M, Stierle M, Fischer B, Brino L, Devys D, Tora L. 2013. The human TREX-2 complex is stably associated with the nuclear pore basket. J Cell Sci 126: 2656–2667. 10.1242/jcs.118000 23591820

[GAD353594ABBC49] Wang Q, Zhang L, Lynn B, Rymond BC. 2008. A BBP-Mud2p heterodimer mediates branchpoint recognition and influences splicing substrate abundance in budding yeast. Nucleic Acids Res 36: 2787–2798. 10.1093/nar/gkn144 18375978 PMC2377449

[GAD353594ABBC50] Wegener M, Müller-McNicoll M. 2018. Nuclear retention of mRNAs - quality control, gene regulation and human disease. Semin Cell Dev Biol 79: 131–142. 10.1016/j.semcdb.2017.11.001 29102717

[GAD353594ABBC51] Weick EM, Puno MR, Januszyk K, Zinder JC, DiMattia MA, Lima CD. 2018. Helicase-dependent RNA decay illuminated by a cryo-EM structure of a human nuclear RNA exosome-MTR4 complex. Cell 173: 1663–1677.e21. 10.1016/j.cell.2018.05.041 29906447 PMC6124691

[GAD353594ABBC52] Wickham H, Averick M, Bryan J, Chang W, D'Agostino McGowan L, François R, Grolemund G, Hayes A, Henry L, Hester J, 2019. Welcome to the Tidyverse. J Open Source Softw 4: 1686. 10.21105/joss.01686

[GAD353594ABBC53] Wickramasinghe VO, Andrews R, Ellis P, Langford C, Gurdon JB, Stewart M, Venkitaraman AR, Laskey RA. 2014. Selective nuclear export of specific classes of mRNA from mammalian nuclei is promoted by GANP. Nucleic Acids Res 42: 5059–5071. 10.1093/nar/gku095 24510098 PMC4005691

[GAD353594ABBC54] Wilmes GM, Bergkessel M, Bandyopadhyay S, Shales M, Braberg H, Cagney G, Collins SR, Whitworth GB, Kress TL, Weissman JS, 2008. A genetic interaction map of RNA-processing factors reveals links between Sem1/Dss1-containing complexes and mRNA export and splicing. Mol Cell 32: 735–746. 10.1016/j.molcel.2008.11.012 19061648 PMC2644724

[GAD353594ABBC55] Xiao SH, Manley JL. 1997. Phosphorylation of the ASF/SF2 RS domain affects both protein-protein and protein-RNA interactions and is necessary for splicing. Genes Dev 11: 334–344. 10.1101/gad.11.3.334 9030686

[GAD353594ABBC56] Xie Y, Clarke BP, Xie D, Mei M, Bhat P, Hill PS, Angelos AE, Çağatay T, Haider M, Collier SE, 2025. Structures and mRNP remodeling mechanism of the TREX-2 complex. Structure 33: 566–582.e6. 10.1016/j.str.2024.12.019 39862860 PMC11890942

[GAD353594ABBC57] Zenklusen D, Vinciguerra P, Wyss JC, Stutz F. 2002. Stable mRNP formation and export require cotranscriptional recruitment of the mRNA export factors Yra1p and Sub2p by Hpr1p. Mol Cell Biol 22: 8241–8253. 10.1128/MCB.22.23.8241-8253.2002 12417727 PMC134069

[GAD353594ABBC58] Zhou Y, Zhu J, Schermann G, Ohle C, Bendrin K, Sugioka-Sugiyama R, Sugiyama T, Fischer T. 2015. The fission yeast MTREC complex targets CUTs and unspliced pre-mRNAs to the nuclear exosome. Nat Commun 6: 7050. 10.1038/ncomms8050 25989903 PMC4455066

[GAD353594ABBC59] Zinder JC, Lima CD. 2017. Targeting RNA for processing or destruction by the eukaryotic RNA exosome and its cofactors. Genes Dev 31: 88–100. 10.1101/gad.294769.116 28202538 PMC5322736

