# Supplementary material for: Evolutionarily conserved spliceosome–exosome pathway in nuclear mRNA surveillance
Source: Genes Dev. 2026 Jul 1;40(13-14):1119–32. doi: 10.1101/gad.353594.125 (PMC13322074; doi:10.1101/gad.353594.125)
Supplement: Supplement 1 [file Supplemental_Data_Abbas_353594.pdf]

Supplemental Materials and Methods

Bacterial Expression and Purification

6xHis-GST-3C-LENG8(501-800) (LENG8$_{core}$), 6xHis-GST-3C-LENG8$_{core}$ D730R, 6xHis-GST-3C-LENG8(550-800)-GS-RRP1B(742-758) (LENG8$_{core\Delta}$-RRP1B$_{L}$), and 6xHis-GST-3C-GANP(598-998) (GANP$_{core}$) were co-expressed with untagged PCID2-DSS1 in *Escherichia coli* BL21 STAR (DE3) pRARE cells.

6xHis-MBP-3C-RRP1B(742-758) (RRP1B$_{L}$), 6xHis-MBP-3C-RRP1B$_{L}$ F757A F758A, 6xHis-MBP-3C-RRP1B$_{L}$ R751D, RRP1B$_{L}$-MBP, LENG8(34-59) (LENG8$_{U}$) -TS-3C-GST-6xHis, LENG8(34-59) A42D-TS-3C-GST-6xHis, LENG8(34-59) W39A L46A-TS-3C-GST-6xHis, and pEC-K-Aly/REF(1-29-GST-229-257) were expressed in *Escherichia coli* BL21 STAR (DE3) pRARE cells.

6xHis-GST-3C-UAP56, 6xHis-GST-3C-FLAG-UAP56, and 6xHis-GST-3C-FLAG-UAP56 D12R Y13A D15R were expressed in *Escherichia coli* Gold (DE3) pLysS cells.

All proteins were expressed overnight at 18°C.

For all the constructs with a 6xHis tag, cells were resuspended in lysis buffer (50 mM sodium phosphate, 250 mM NaCl, 30 mM imidazole, 2.5 mM β-mercaptoethanol), lysed by sonication, and cleared by centrifugation at 75,600 x*g* for 30 minutes. The proteins were purified using Nickel-based affinity chromatography with elution in 500 mM imidazole, followed by tag cleavage with 3C protease overnight. The proteins were then purified using ion-exchange chromatography (either Heparin or anion exchange) and elution occurred over an increasing NaCl gradient. The fractions containing protein were concentrated and run over a Superdex 200 HiLoad column or Superdex 75 HiLoad column using size-exclusion buffer (20 mM HEPES pH 7.5, 250 mM NaCl, glycerol (v/v) 5%, 2 mM dithiothreitol).

For the UAP56 constructs, these buffers were supplemented with 5 mM magnesium acetate.

In the case of RRP1B$_L$-MBP, cells were lysed in 50 mM potassium phosphate pH 8.0, 250 mM NaCl, 2 mM dithiothreitol buffer and subjected to amylose resin-based affinity chromatography with elution occurring with 25 mM maltose, before subsequent size-exclusion chromatography. In the case of Aly/REF(1-29-GST-229-257), cells were lysed in 20 mM HEPES pH 7.5, 150 mM NaCl, 2 mM dithiothreitol buffer and subjected to glutathione resin-based affinity chromatography with elution occurring with 10 mM glutathione, before subsequent size-exclusion chromatography.

Sem1 was cloned as an N-terminal His–Thioredoxin fusion protein with a 3C protease–cleavable site. Sac3(86–565) (Sac3$_{core}$) and Thp1 were cloned into vectors as N-terminal His–SUMO fusion proteins with cleavage sites for SENP2 protease, while Csn12 and Thp3(186–471) (Thp3$_{core}$) were cloned as N-terminal His-tagged fusion proteins containing a 3C protease–cleavable site. Sem1 was expressed separately in BL21(DE3) STAR pRARE while Sac3/Thp1 and Csn12/Thp3 were co-expressed in Rosetta DE3. Sub2 (1-30) and Sub2 (1-30 E11R, Y12A, D14R) were cloned as N-terminal His–Thioredoxin tagged fusion proteins containing a 3C protease–cleavable site and a C-Terminal TwinStrep tag and were expressed in BL21(DE3) STAR pRARE. For all the yeast protein purifications we used the same purification scheme: cells were mixed and resuspended in lysis buffer (50 mM NaH_2PO_4 pH 7.5, 150 mM NaCl, 40 mM imidazole, 5 mM β-mercaptoethanol) and lysed by sonication. The complex was purified using Nickel-based affinity chromatography followed by tag cleavage with either SENP2 or 3C protease at 4°C for 4 hours. Upon dilution, the complex was further purified with an ion-exchange chromatography column at pH 8.0 (Heparin) and a size-exclusion chromatography (Superdex 200) in size-exclusion buffer (Tris 20 mM pH 8.0, 150 mM NaCl and 2 mM dithiothreitol). We reconstituted both Sac3/Thp1/Sem1 and

Thp3/Csn12/Sem1 by mixing both complexes with a slight excess of Sem1 (1.2X) and running a final size-exclusion chromatography (Superdex 200) in size-exclusion buffer.

Yeast strain engineering

C-terminal tags (TwinStrep-3C-protein A or TurboID-HA) were introduced in *S. cerevisiae* (BY4741 background) at genomic loci using standard yeast genetics techniques. A $(GS)_5$ linker was engineered to separate the tag from the last amino acid in the protein sequence. Integration was confirmed by PCR and Western blot.

Quantitative Mass Spectrometry

Proteins bound to magnetic beads or TCA-pelleted proteins were denatured by adding 50 µL of SDC buffer containing 1% SDC, 40 mM CAA, 10 mM TCEP, and 100 mM Tris (pH 8.0). The samples were incubated for 30 min at 37 °C, followed by overnight digestion at 37 °C with 0.5 µg trypsin (Promega). The resulting peptide solution was acidified with TFA (Merck) to a final concentration of 1% and directly loaded onto Evotips.

In-gel band digestion was performed by washing the gel bands of interest three times with 150 µL of destaining buffer (25 mM ammonium bicarbonate, 50% ethanol) and then dehydrated twice with 150 µL of 100% ethanol. After removing the ethanol, the gel pieces were dried by vacuum centrifugation. Next, 50 µL of digestion buffer (50 mM ammonium bicarbonate, 10 ng/µL trypsin, 0.01% ProteaseMax) was added. The mixture was incubated on ice for 20 minutes, after which 50 µL of ammonium bicarbonate buffer (50 mM containing 0.01% ProteaseMax) was added. The gel pieces were then incubated overnight at 37°C. Peptides in

the supernatant were collected, and additional peptides were extracted from the gel pieces by incubating them at 25°C in 100 μL of extraction buffer (3% TFA, 30% acetonitrile), followed by centrifugation and collection of the supernatant. The gel pieces were then dehydrated at 25 °C in 100 μL of 100% acetonitrile, and the resulting supernatant was combined with the previous extracts. Acetonitrile was removed by vacuum centrifugation, and 50 μL of 2 M Tris-HCl containing 10 mM TCEP and 40 mM CAA was added. After a 30-minute incubation at 37 °C, the peptides were acidified to 1% TFA and loaded onto Evotips.

Peptides were eluted from the Evotips onto a PepSep C18 column (15 cm × 150 μm, 1.5 μm particle size) using the Evosep One HPLC system. The column was maintained at 50 °C, and peptide separation was achieved using the 30 SPD method. Eluted peptides were directly ionized and introduced into a timsTOF Pro mass spectrometer via electrospray ionization. Data acquisition was performed in data-independent acquisition (DIA) PASEF mode via timsControl. Mass spectrometry covered a scan range of 100–1700 m/z, and ion mobility ranged from 1/K0 = 0.70 to 1.30 Vs·cm^2. The dual TIMS analyzer utilized equal ion accumulation and ramp times of 100 ms each, with a spectra rate of 9.52 Hz. For DIA-PASEF scans, the mass scan range was 350.2–1199.9 Da, and ion mobility ranged from 1/K0 = 0.70 to 1.30 Vs·cm^2. Collision energy was linearly ramped based on ion mobility, from 45 eV at 1/K0 = 1.30 Vs·cm^2 to 27 eV at 1/K0 = 0.85 Vs·cm^2. A total of 42 DIA-PASEF windows were acquired per TIMS scan, with switching precursor isolation windows, resulting in an estimated cycle time of 2.21 seconds.
Raw data were processed with Spectronaut version 20 in directDIA+ (library-free) mode. Spectra were searched against a predicted Homo sapiens database (UniProt SwissProt, canonical; UP000005640, downloaded February 2024) using standard settings. Cysteine carbamidomethylation was defined as a fixed modification, while methionine oxidation and

protein N-terminal acetylation were set as variable modifications. The protease was specified as Trypsin/P.

Cross-linking Mass Spectrometry

TCA-precipitated, BS^3-cross-linked samples were solubilized by addition of 8 M urea in 50 mM Tris and sonication using a Bioruptor Plus system 10 times for 30 sec at high intensity. For reduction and alkylation, 10 mM TCEP and 40 mM CAA were added. After incubation for 20 min at 37°C, samples were diluted 1:3 with water and digested overnight at 37°C by addition of 0.5 µg of LysC and 1 µg of Trypsin. Next, the solution was acidified with TFA to a final concentration of 1% and approximately 400 ng of peptide material was loaded on Evotips.

Peptides were then eluted from the Evotips onto a 15-cm PepSep C18 column (15 cm × 150 µm, 1.5 µm particle size) using the Evosep One HPLC system, employing the 30 samples per day (SPD) method. Mass spectrometry analysis was carried out on an Orbitrap Exploris 480 equipped with a FAIMS Pro interface set to standard resolution, with compensation voltages of −50−60 V and −45−55 V, operated in data-dependent acquisition mode. Full MS scans were collected from m/z 300 to 1650 Th at a resolution of 60,000 (at m/z 200 Th). The top 15 most intense precursor ions were selected for fragmentation using stepped higher-energy C-trap dissociation (HCD) at normalized collision energies of 19, 27, and 35. MS2 spectra were acquired with a resolution of 30,000 (at m/z 200 Th) across a dynamic m/z range. Normalized automatic gain control (AGC) targets were set to 300% for MS1 and 100% for MS2, with a maximum injection time of 25 ms for MS1 and "auto" for MS2. Ions with a charge state of +2 were excluded to prioritize cross-linked precursors.

The acquired raw data were processed using pLink3. Carbamidomethylation on cysteines was set as fixed modification, oxidation on methionine was set as variable modification and Trypsin/P was specified as protease, and up to two missed cleavages were allowed. Filtering at 1% false discovery rate (FDR) was applied.

Abbas et al., Supplemental Figure S1

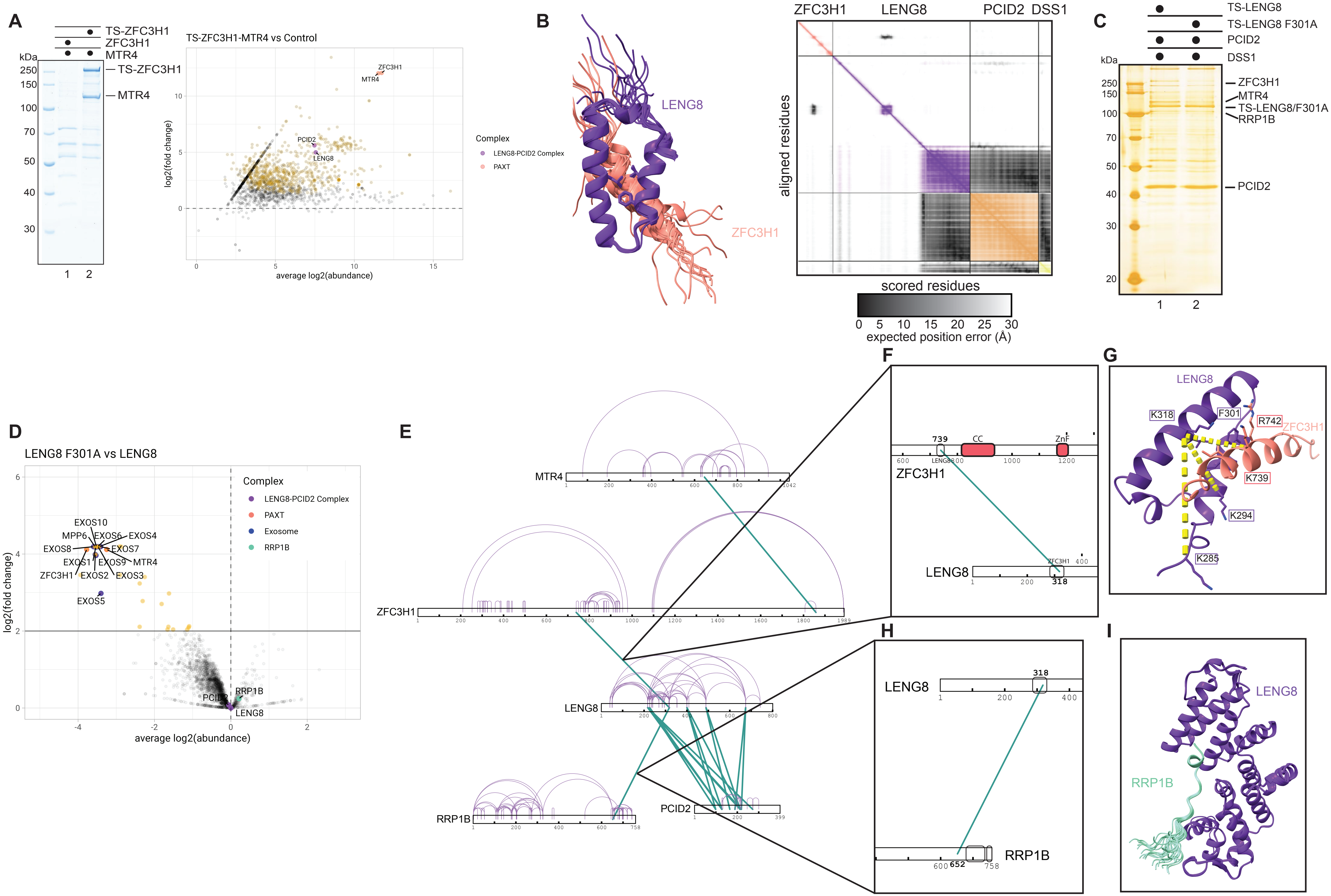

Supplemental Figure S1. LENG8 recruits the nuclear exosome through interaction with ZFC3H1

(A) Affinity purification from HEK 293T cells expressing TS-ZFC3H1-MTR4 (left panel), showing enrichment of LENG8 and PCID2 in a Coomassie-stained SDS gel analysis and mass spectrometry plot-based enrichment analysis (right) of triplicate repeats. Statistically significant fold changes at p-value ≤ 0.01 are colored in gold.

(B) AlphaFold-Multimer version 2.3.1 prediction depicting 16/25 superimposed models of the LENG8 (purple) and ZFC3H1 (salmon) interaction involving the three-helix bundle and conserved hydrophobic residues, and the respective predicted aligned error (PAE) plot colored according to protein.

(C) Silver-stained gel of an affinity purification of TS-tagged wild type and F301A mutant LENG8 from HEK 293T cells. Data is a replication of that shown in Figure 1C but with alternative staining to enhance visibility.

(D) Volcano plot of the mass spectrometry plot-based enrichment analysis shown in Figure 1C. Statistically significant fold changes at p-value ≤ 0.01 are colored in gold with a horizontal line drawn at p-value ≤ 0.01.

(E) Cross-links between MTR4, ZFC3H1, LENG8, RRP1B, and PCID2, present in all three replicates of the TS-LENG8 XL-MS sample, are mapped on linearized schematics of the proteins. Intraprotein cross-links are represented in purple, and interprotein cross-links in green.

(F) Zoom-in view of the cross-link between ZFC3H1 Lys739 and LENG8 Lys318 shown on a linear protein representation with domain architecture indicated.

(G) Structural representation of the cross-link (yellow) between ZFC3H1 Lys739 and LENG8 Lys318 on the AlphaFold prediction shown in Suppl. Fig. S1B. Key residues of LENG8 (purple) and ZFC3H1 (salmon) are indicated.

(H) Zoom-in view of the cross-link between LENG8 Lys318 and RRP1B Lys652 shown on a linear protein representation with domain architecture indicated.

(I) AlphaFold-Multimer version 2.3.1 prediction depicting 25/25 superimposed models of LENG8 (purple) showing the interaction with the extreme C-terminus of RRP1B (residues 742-758) (green).

Abbas et al., Supplemental Figure S2

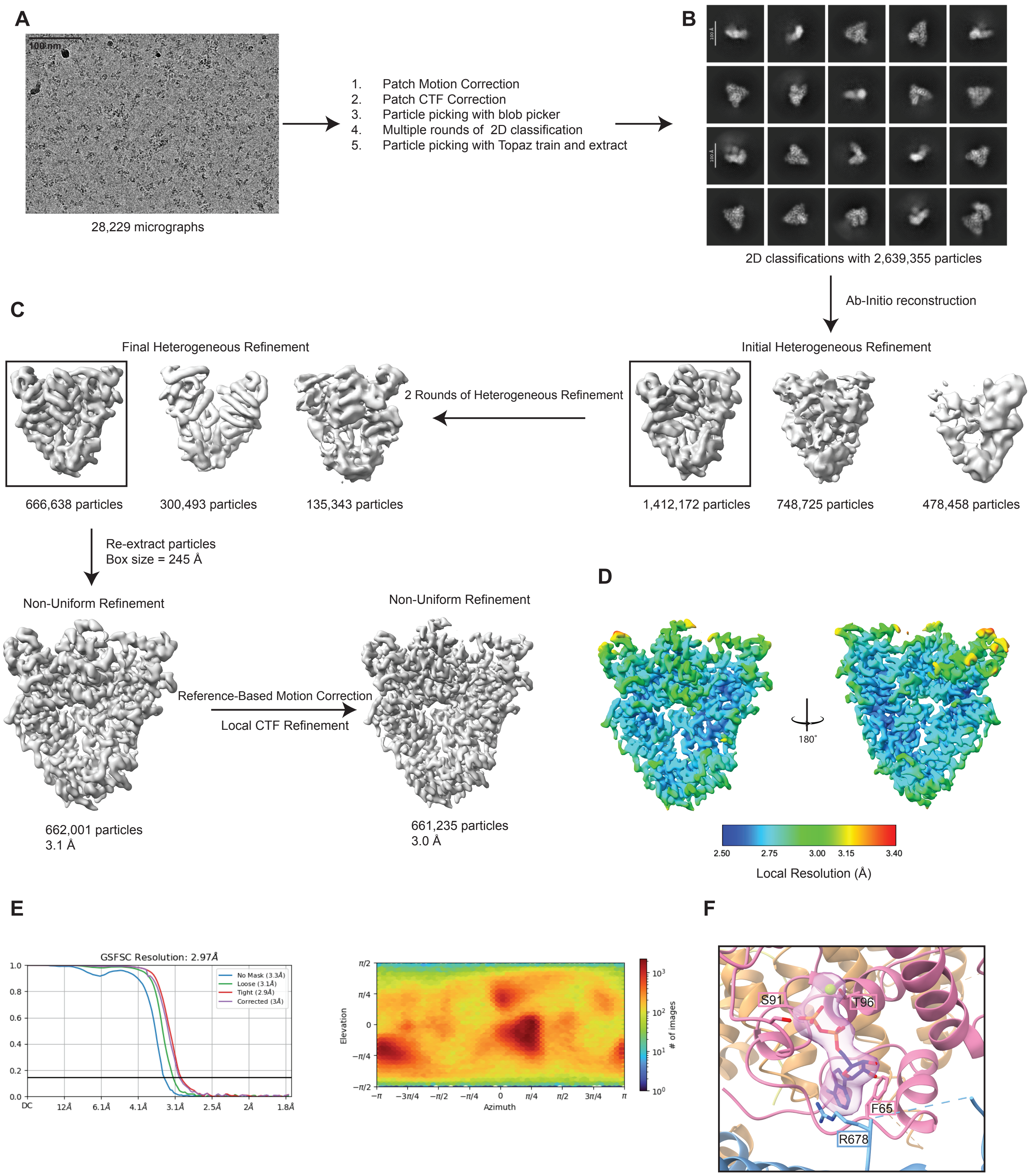

Supplemental Figure S2. Cryo-EM analysis of $GANP_{core}$-PCID2-DSS1-UAP56

(A) Representative cryo-EM micrograph and initial processing steps. Scale bar represents 100 nm.

(B) Representative 2D class averages.

(C) Cryo-EM data processing scheme. Three *ab-initio* reconstructions were generated from 60,000 particles. 2,639,355 particles were sorted into these three *ab-initio* reconstructions and subsequently, multiple rounds of heterogeneous refinement cleaned the particle stack of the highest resolution density (shown in a black box). The cleaned particle stack was re-extracted using a box size of 245 Å, yielding a resolution of 3.1 Å after non-uniform refinement, which was improved to 3.0 Å following reference-based motion correction and local CTF refinement. All maps shown are unsharpened maps obtained after refinements.

(D) Local resolution estimate of the final cryo-EM map, shown in two different orientations, related by 180° rotation.

(E) Gold-standard Fourier shell correlation (GSFSC) curve (left panel) and an angular distribution plot obtained from cryoSPARC (right panel).

(F) Zoom-in view of the cryo-EM structural model of $GANP_{core}$-PCID2-DSS1-UAP56 focusing on the nucleotide binding site. Magnesium (green) and ADP are both shown within a transparent pink density. Residues involved in coordinating the magnesium and β-phosphate (UAP56 S91, T96) and residues involved in the interaction with the adenosine (UAP56 F65 and GANP R678) are labelled. UAP56 is colored in pink, GANP in blue, and PCID2 in orange.

Abbas et al., Supplemental Figure S3

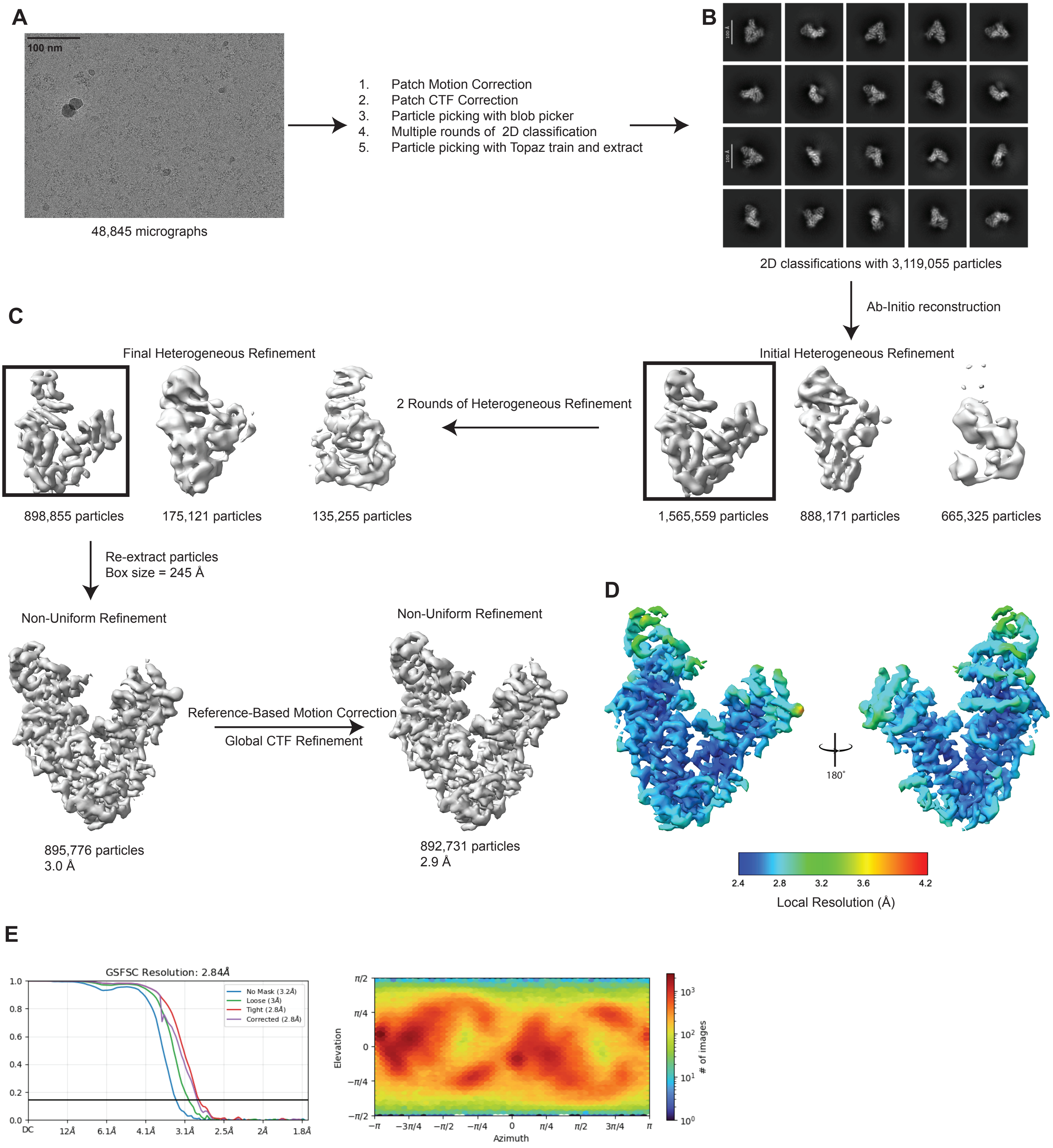

Supplemental Figure S3. Cryo-EM analysis of LENG8$_{core\Delta}$-RRP1B$_L$-PCID2-DSS1-UAP56

(A) Representative cryo-EM micrograph and initial processing steps. Scale bar represents 100 nm.

(B) Representative 2D class averages.

(C) Cryo-EM data processing scheme. Three *ab-initio* reconstructions were generated from 57,138 particles. 3,119,055 particles were sorted into these three *ab-initio* reconstructions and subsequently, multiple rounds of heterogeneous refinement cleaned the particle stack of the highest resolution density (shown in a black box). The cleaned particle stack was re-extracted using a box size of 245 Å, yielding a resolution of 3.0 Å after non-uniform refinement, which was improved to 2.9 Å following reference-based motion correction and global CTF refinement. All maps shown are unsharpened maps obtained after refinements.

(D) Local resolution estimate of the final map, shown in two different orientations, related by 180° rotation.

(E) Gold-standard Fourier shell correlation (GSFSC) curve (left panel) and an angular distribution plot obtained from cryoSPARC (right panel).

Abbas et al., Supplemental Figure S4

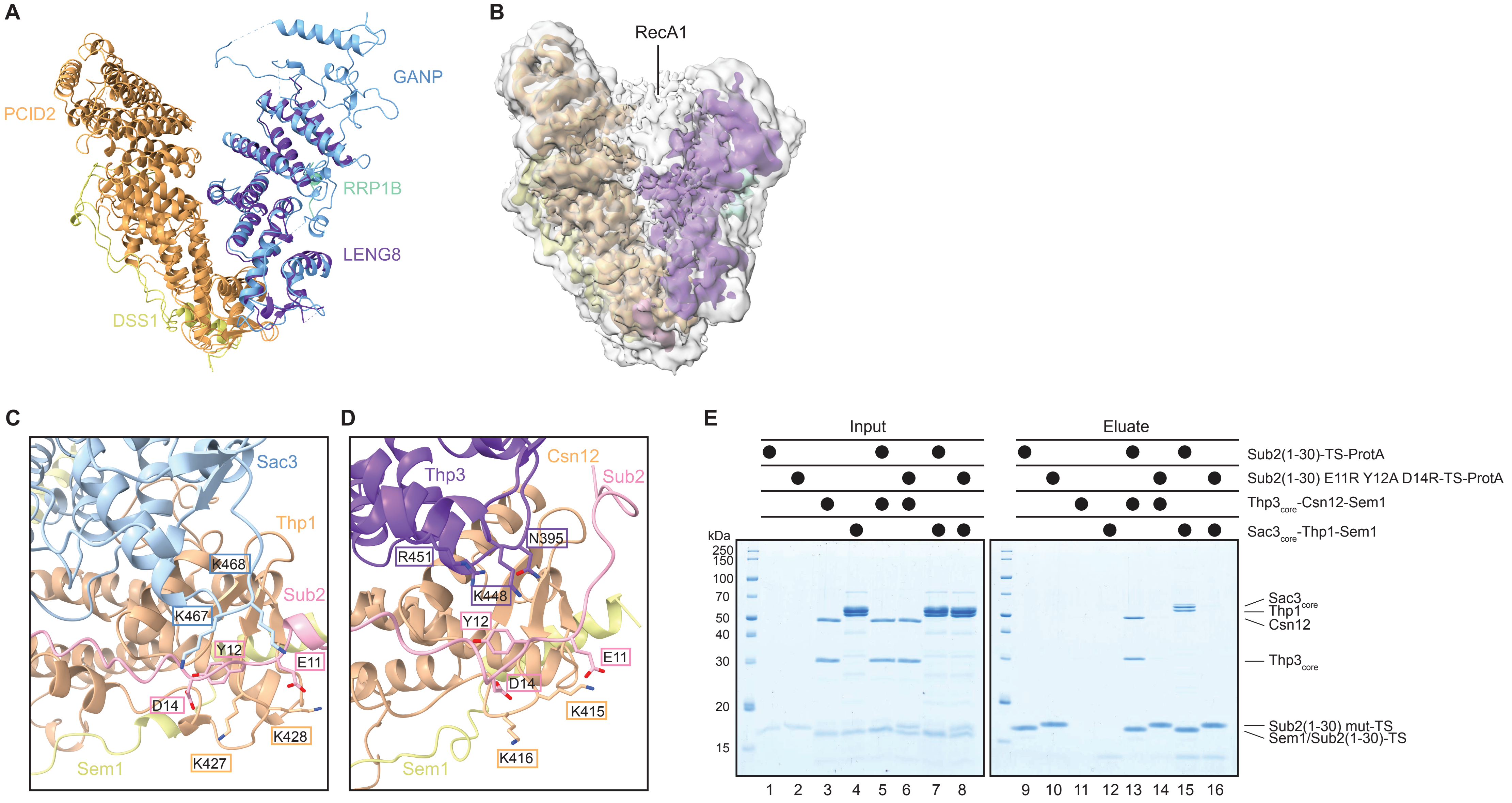

Supplemental Figure S4. The LENG8-PCID2 and GANP-PCID2 complexes exhibit similar binding to UAP56

(A) Superimposition of the LENG8$_{core\Delta}$-RRP1B$_{L}$-PCID2-DSS1-UAP56 complex and GANP$_{core}$-PCID2-DSS1-UAP56 complex structures showing the shorter LENG8 PCI domain compared to the GANP PCI domain. UAP56 was hidden from view to enable an unimpeded view of the PCI domains. DSS1 is shown in yellow, PCID2 in orange, LENG8 in purple, and GANP in blue.

(B) LENG8$_{core\Delta}$-RRP1B$_{L}$-PCID2-DSS1-UAP56 map contoured at a lower level superimposed on the reconstruction shown in Fig. 2H. At this lower contour level, the highly flexible UAP56 RecA1 domain becomes visible as depicted by the gray transparent density.

(C) Zoom-in view of an AlphaFold-Multimer version 2.3.1 prediction showing the N-terminal motif of Sub2 interacting with Sac3-Thp1. Key residues in the interaction are depicted. Sac3 is shown in blue, Thp1 in orange, Sem1 in yellow, and Sub2 in pink.

(D) Zoom-in view of an AlphaFold-Multimer version 2.3.1 prediction showing the N-terminal motif of Sub2 interacting with Thp3-Csn12. Key residues in the interaction are depicted. Thp3 is shown in purple, Csn12 in orange, Sem1 in yellow, and Sub2 in pink.

(E) Coomassie-stained SDS gel analysis of Sub2(1-30)-TS pull-down assays. Association between Sub2 with either the Thp3 complex or the Sac3 complex was abolished when Sub2(1-30) containing a triple mutant of E11R Y12A D14R was used for pull-down (compare lanes 13 and 15 to lanes 14 and 16).

Abbas et al., Supplemental Figure S5

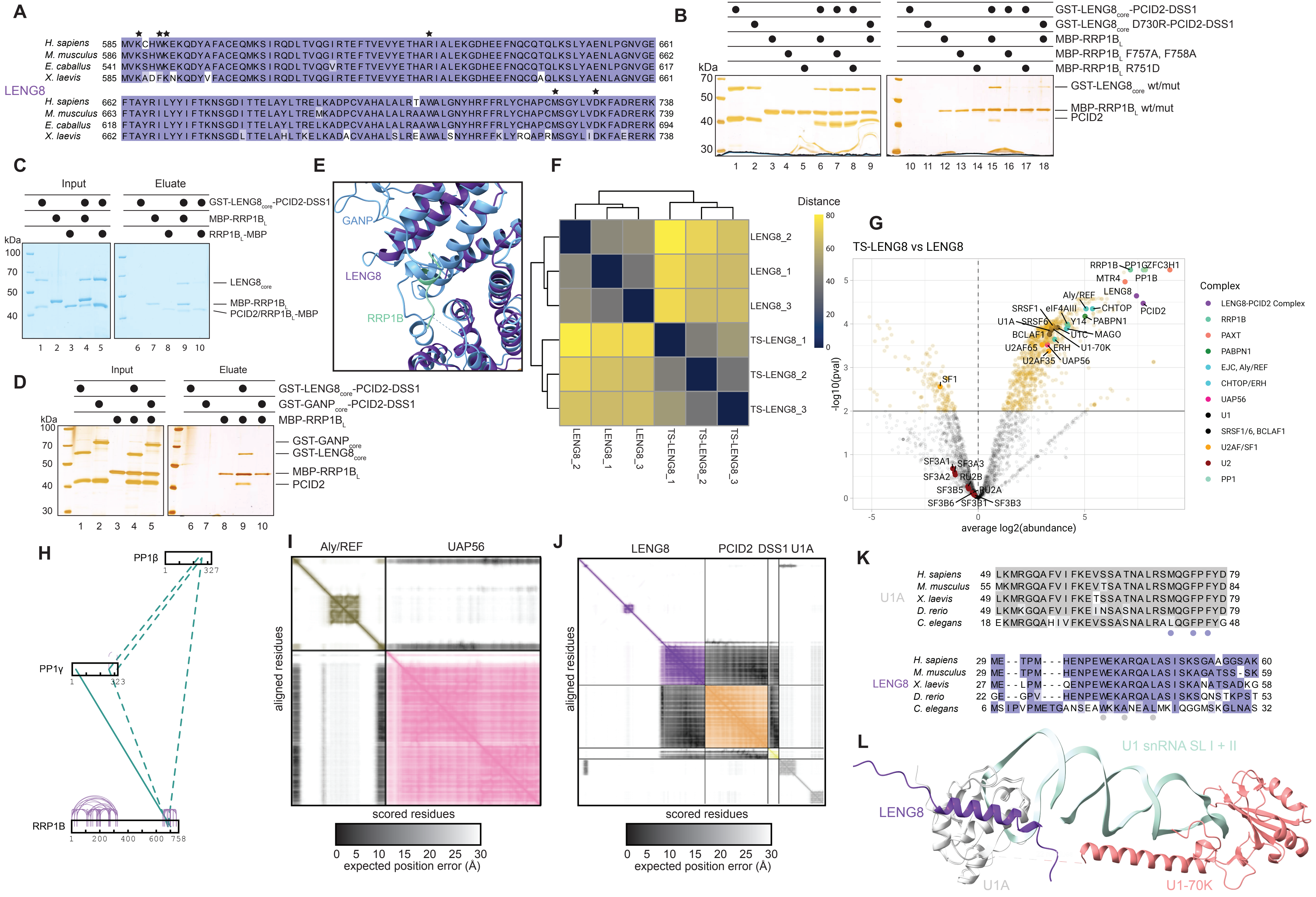

Supplemental Figure S5. LENG8-PCID2 associates with RRP1B and mRNPs containing early splicing factors

(A) Multiple sequence alignment of LENG8. Residues involved in the interaction with RRP1B are indicated by stars.

(B) Silver-stained SDS gel analysis of MBP-RRP1B$_L$ pull-down assays. Data is a replication of that shown in Figure 3C but with alternative staining to enhance visibility.

(C) Coomassie-stained SDS gel analysis of MBP-RRP1B$_L$ pull-down assays. Pull-down of the LENG8$_{core}$ complex was abolished with C-terminally, rather than N-terminally, MBP-tagged RRP1B$_L$ (compare lane 9 to lane 10).

(D) Silver-stained SDS gel analysis of MBP-RRP1B$_L$ pull-down assays of LENG8-PCID2 complex and GANP-PCID2 complex. Data is a replication of that shown in Figure 3D but with alternative staining to enhance visibility.

(E) Superimposition of the LENG8$_{core\Delta}$-RRP1B$_L$-PCID2-DSS1-UAP56 complex and GANP$_{core}$-PCID2-DSS1-UAP56 complex structures showing the GANP helix blocking the putative RRP1B binding site. RRP1B is colored in green, LENG8 in purple, and GANP in blue.

(F) Heatmap of the sample-to-sample Euclidean distances using log-transformed mass spectrometry quantity data shown in Fig. 4A.

(G) Volcano plot of the mass spectrometry plot-based enrichment analysis shown in Figure 4A. Statistically significant fold changes at p-value ≤ 0.01 are colored in gold with a horizontal line drawn at p-value ≤ 0.01.

(H) Cross-links between RRP1B, PP1β, and PP1γ, present in all three replicates of the TS-LENG8 XL-MS sample, are mapped on linearized schematics of the proteins. Intraprotein cross-links are represented in purple, and interprotein cross-links in green.

(I) Predicted aligned error (PAE) plot, colored according to protein, of the AlphaFold-Multimer version 2.3.1 prediction of the Aly/REF interaction with UAP56 shown in Figure 4B and C.

(J) Predicted aligned error (PAE) plot, colored by chain, of the AlphaFold-Multimer version 2.3.1 prediction shown in Fig. 4E.

(K) Multiple sequence alignments of U1A (gray) and LENG8 (purple). The specific interacting residues are indicated underneath with colored dots.

(L) Superimposition of the AlphaFold-Multimer version 2.3.1 prediction of LENG8 (purple) and U1A (gray) on the structure of U1-70K (salmon) in complex with U1 snRNA stem-loop 1 and U1A RRM in complex with stem-loop 2 (PDB: 4PKD, (Kondo et al. 2015)), showing that the LENG8-U1A interaction is compatible with the U1A interaction with the U1 snRNA (light blue).

Kondo Y, Oubridge C, van Roon AM, Nagai K. 2015. Crystal structure of human U1 snRNP, a small nuclear ribonucleoprotein particle, reveals the mechanism of 5' splice site recognition. *Elife* **4**.

Abbas et al., Supplemental Figure S6

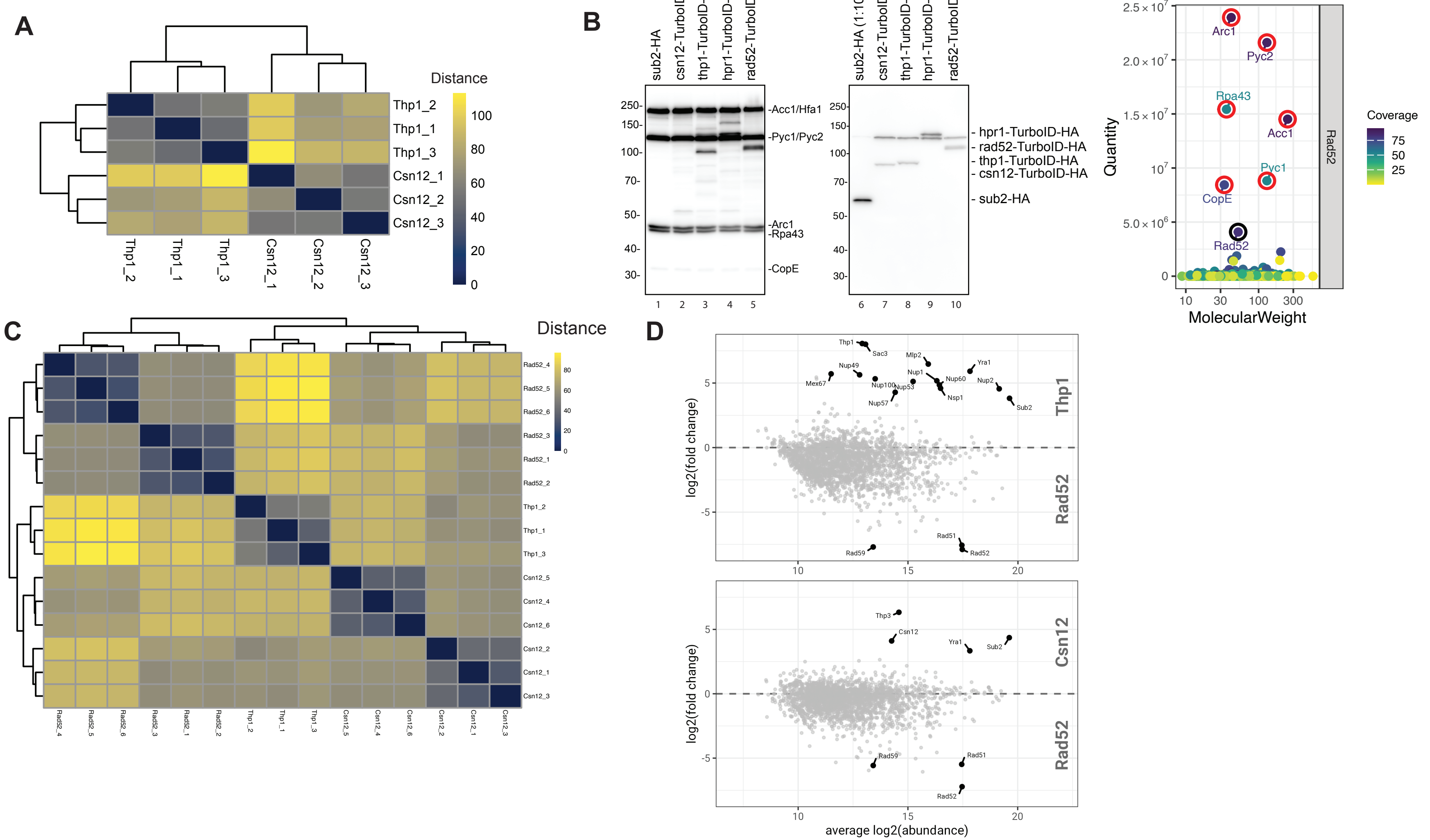

Supplemental Figure S6. Thp3-Csn12 transiently associates with nuclear exosome subunits

(A) Heatmap of the sample-to-sample Euclidean distances using log-transformed mass spectrometry quantity data shown in Fig. 5A.

(B) Validation of TurboID yeast strains.

For each strain, the target protein is C-terminally tagged at the endogenous locus with a sequence coding for TurboID followed by an HA-tag. Total protein content from each yeast strain was extracted in SDS-PAGE loading dye after NaOH treatment according to proximity labeling methods. 1% of the extract before biotin removal was separated on a 10% SDS-PAGE and transferred to a PVDF membrane before labeling with Streptavidin-HRP, or with an anti-HA antibody followed by anti-mouse-HRP secondary antibody. Proteins were visualized using an enhanced chemiluminescence kit (left). For the control strain where Sub2 is tagged with HA only, 0.1% total extract was loaded on the gel to account for the 10-to-50-fold higher abundance of this protein compared to the other targets. Five bands corresponding to endogenously biotinylated proteins are visible in all strains. Some of these proteins were previously characterized (Acc1, Hfa1, Pyc1, Pyc2, Arc1). To our knowledge, Rpa43 and CopE, which we identified by mass spectrometry (right) after purification and in-gel band digestion, has not yet been identified as bearing a biotin modification.

(C) Heatmap of the BioID sample-to-sample Euclidean distances using log-transformed mass spectrometry quantity data shown in Fig. 5B.

(D) MS plot-based enrichment analysis of pull-downs performed in at least triplicate from BioID strains Thp1 and Csn12 compared to the negative control of Rad52.

**Data Collection Parameters and Processing**		
Dataset	**LENG8$_{C\Delta}$-RRP1B$_{L}$-PCID2-DSS1-UAP56**	**GANP$_{C}$-PCID2-DSS1-UAP56**
Microscope	FEI Titan Krios G2	
Voltage [kV]	300	
Camera	Gatan K3	
Magnification	105,000x	
Pixel size [Å]	0.8512	
Electron exposure [e^-/Å^2]	59.0	61.8
Target defocus range [µm]	0.5 – 2.0	
Number of movies	48,845	28,229
Initially selected particles	3,119,055	2,639,355
Final number of particles	892,731	661,235
Resolution [Å]	2.9	3.0
Local resolution range [Å]	2.4 – 4.1	2.6 – 6.6
Sharpening B-factor [Å^2]	-160.8	-154.4
**Refinement**		
Number of non-hydrogen atoms	5404	8254
Residues (protein/nucleotide)	671/0	1024
Ligands	0	Magnesium, ADP
CC_{box}, CC_{mask}, CC_{volume}	0.90, 0.89, 0.88	0.88, 0.87, 0.87
CC for individual chains (Magnesium = A, UAP56 = B, LENG8$_C$ = C_L, GANP$_C$ = C_G, PCID2 = D, DSS1 = E, RRP1B = F	B = 0.836, C_L = 0.874, D = 0.872, E = 0.862, F = 0.842	A = 0.761, B = 0.818, C_G = 0.851, D = 0.846, E = 0.820
Resolution$_{FSC\ map\ vs.\ model\ (0/0.143/0.5)}$ [Å]	2.8/2.8/3.0	2.9/2.9./3.1
r.m.s. deviations		
Bond lengths [Å]	0.002	0.002
Bond angles [°]	0.480	0.433
Ramachandran favoured [%]	97.72	98.00
Ramachandran allowed [%]	2.28	2.00
Ramachandran outliers [%]	0.00	0.00
MolProbity score	1.22	0.93
Clash score	3.74	1.77
Rotamer outliers [%]	0.00	0.00

REAGENT or RESOURCE	SOURCE	IDENTIFIER
**Antibodies**		
Mouse monoclonal anti-HA Tag 1:1000	Covance	MMS-101R
HRP-conjugated streptaviding anti-biotin 1:3333	Thermo Fischer Scientific	S911
Mouse monoclonal anti-ProteinA	Sigma-Aldrich	P2921
**Bacterial strains**		
Escherichia coli BL21 (DE3) STAR pRARE	Invitrogen, transformed with in-lab plasmid pRARE	C601003
Escherichia coli Omnimax	Invitrogen	C854003
Escherichia coli BL21 (DE3) pRARE	NEB, transformed with in-lab plasmid pRARE	C2527H
Escherichia coli Gold (DE3) pLysS	Agilent	230134
Escherichia coli Rosetta (DE3)	Novagen	70954
**Yeast strains**		
Saccharomyces cerevisiae BY4741	Euroscarf	Y00000
**Chemicals and peptides**		
3xFLAG peptide	MPIB Bioorganic Chemistry & Biophysics core facility	N/A
**Deposited data**		
LENG8-PCID2-DSS1 complex bound to UAP56 and RRP1B	This paper	EMDB: 55617, PDB: 9T6L
GANP-PCID2-DSS1 complex bound to UAP56	This paper	EMDB: 55619, PDB: 9T6N
**Recombinant DNA**		
pEC-K-6xHis-GST-3C-LENG8(501-800)	This study	N/A
pEC-K-6xHis-GST-3C-LENG8(550-800)-GS-RRP1B(742-758)	This study	N/A
pEC-K-6xHis-GST-3C-GANP(598-998)	This study	N/A
pCDF-Duet-1-PCID2-DSS1	This study	N/A
pEC-A-6xHis-GST-UAP56	This study	N/A
pEC-A-6xHis-GST-UAP56 (D12R Y13A D15R)	This study	N/A
pEC-K-6xHis-GST-3C-LENG8(501-800) D730R	This study	N/A
pEC-A-6xHis-GST-3C-FLAG-UAP56	This study	N/A
pEC-K-6xHis-MBP-HT-RRP1B(742-758)	This study	N/A
pEC-K-6xHis-MBP-HT-RRP1B(742-758) F757A, F758A	This study	N/A
pEC-K-6xHis-MBP-HT-RRP1B(742-758) R751D	This study	N/A
pEC-K-RRP1B(742-758)-MBP	This study	N/A

pEC-K-LENG8(34-59)-TS-3C-GST-6xHis	This study	N/A
pEC-K-LENG8(34-59) A42D-TS-3C-GST-6xHis	This study	N/A
pEC-K-LENG8(34-59) W39A L46A-TS-3C-GST-6xHis	This study	N/A
pEC-A-6xHis-MBP-3C-FLAG-U1A(1-99)	This study	N/A
pEC-A-6xHis-MBP-3C-FLAG-U1A(1-99) F75D	This study	N/A
pEC-K-3C-TRX-Sem1	This study	N/A
pEC-K-6xHis-SUMO-Thp1	This study	N/A
pEC-A-6xHis-GST-3C-Csn12	This study	N/A
pEC-A-6xHis-SUMO-Sac3(86-565)	This study	N/A
pEC-K-6xHis-GST-3C-Thp3(186-471)	This study	N/A
pEC-K-6xHis-TRX-Sub2(1-30)-ProtA-TS	This study	N/A
pEC-K-6xHis-TRX-Sub2(1-30) (E11R Y12A D14R)-ProtA-TS	This study	N/A
pEC-K-Aly/REF(1-29-GST-229-257)	This study	N/A
pB-T-LENG8	This study	N/A
pB-T-TS-LENG8	This study	N/A
pB-T-TS-LENG8 F301A	This study	N/A
pB-T-PCID2	This study	N/A
pB-T-DSS1	This study	N/A
pB-T-MTR4	This study	N/A
pB-T-ZFC3H1	This study	N/A
pB-T-TS-ZFC3H1	This study	N/A
**Software and algorithms**		
UCSF ChimeraX v.1.10	Goddard et al.	https://www.cgl.ucsf.edu/chimerax/
AlphaFold2	Jumper et al.	https://github.com/google-deepmind/alphafold
AlphaFold Multimer	Evans et al.	https://github.com/google-deepmind/alphafold

Phenix.refine	Afonine et al.	https://phenix-online.org/
Coot v0.9.8.95	Emsley et al.	https://www2.mrc-lmb.cam.ac.uk/personal/pemsley/coot/
Topaz	Bepler et al.	https://cb.csail.mit.edu/topaz/
cryoSPARC v4.7.0	Punjani et al.	https://cryosparc.com/
R	R Core Team	https://www.r-project.org/
Spectronaut v20	Biognosys	
**Other**		
Superdex 75 pg 16/60	Cytiva	28989333
Superdex 200 Increase 3.2/300	Cytiva	29036232
Superdex 200 pg 16/600	Cytiva	28989335
HisTrap FF 5 ml	Cytiva	17525501
TALON Superflow His-tagged resin	Cytiva	28957502
HiTrap Q HP 5 ml	Cytiva	17115401
HiTrap Heparin HP 5 ml	Cytiva	17040703
StrepTactin	IBA Lifesciences	2-1204-005
Dynabeads™ M-270 Epoxy	Thermo Fisher Scientific	14302D
Dynabeads™ M-270 Streptaviding	Thermo Fisher Scientific	65305
Anti-FLAG® M2 Magnetic Beads	Merck	M8823
Amylose Magnetic Beads	NEB	E8035S
MagStrep® Strep-Tactin®XT beads	IBA Lifesciences	2-5090-010
Dynabeads™ Protein G	Thermo Fisher Scientific	10009D
Zeba dye and Biotin Removal Spin Columns	Thermo Fisher Scientific	A44296
SUPERase·In™ RNase Inhibitor	Thermo Fisher Scientific	AM2694
Amicon Ultra MWCO30	Merck	UFC9030
Amicon Ultra MWCO10	Merck	UFC8010
EDTA-free cOmplete Protease Inhibitor Cocktail	Roche	12352204

Protein LoBind Tubes	Eppendorf	0030108116
ÄKTApurifier	GE Healthcare	N/A
SPEX SamplePrep 6875/6875A Freezer/Mill	SPEX	N/A
PepSep C18 Column	Bruker Daltonics	N/A
Evotips	Evosep	N/A
Orbitrap Exploris 480	Thermo Fisher Scientific	N/A
Evosep One HPLC System	Evosep	N/A
timsTOF Pro Mass Spectrometer	Bruker Daltonics	N/A
Bioruptor Plus System	Diogenode	B01020014
Protein Detective Stain	Biozol	BZL-PD1
Pierce Silver Stain Kit	Thermo Fisher Scientific	24612
